# Supplementary material for: Global warming may increase the burden of obstructive sleep apnea
Source: Nat Commun. 2025 Jun 16;16:5100. doi: 10.1038/s41467-025-60218-1 (PMC12170837; doi:10.1038/s41467-025-60218-1)
Supplement: Supplementary file 1 — Supplementary Information [file 41467_2025_60218_MOESM1_ESM.pdf]

Supplementary material: Global warming may increase the burden of obstructive sleep apnea.

Bastien Lechat[1#], Jack Manners[1], Lucía Pinilla[1], Amy Reynolds[1], Hannah Scott[1], Daniel Vena[2], Sebastien Bailly[4], Barbara Toson[1], Billingsley Kaambwa[3], Robert Adams[1], Jean-Louis Pepin[4], Pierre Escourrou[5], Peter Catcheside[1], Danny J Eckert[1]

[#] Corresponding author

bastien.lechat@flinders.edu.au

Mark Oliphant Building, Level 2, Building A, 5 Laffer Drive, Bedford Park 5042

[1] Adelaide Institute for Sleep Health and FHMRI Sleep Health, College of Medicine and Public Health, Flinders University, Adelaide, Australia

[2] Health Economics Unit, College of Medicine and Public Health, Flinders University, Health Sciences Building, Sturt Road, Bedford Park, SA, 5042, Australia

[3] Univ. Grenoble Alpes, HP2 Laboratory, Inserm U-1300, CHU Grenoble Alpes, 38043 Grenoble, France.

[4] Centre Interdisciplinaire du Sommeil, Paris, France

Contents

Supplementary Results ... 4

Participants ... 4

Supplementary Tables and Figures ... 5

Supplementary methods ... 32

Assessment of weather variables and climate projections ... 32

Statistical analyses ... 33

Supplementary acknowledgments ... 35

References ... 37

List of tables

Table S1: Risk ratio (OR) (95%CI) for nightly obstructive sleep apnea (OSA) and nightly severe OSA at 99th versus 25th percentile of temperature in each location. Demographics, number of participants, and average number of recordings per participant are included for each location. ... 5

Table S2: Projected wellbeing burden and associated cost of warming-related increase in moderate-to-severe obstructive sleep apnea (OSA) prevalence for different countries. Disability-adjusted life years (DALY) rates were calculated for each country in 2000 and 2023, and the % change was calculated. Years of life lost (YLL), years lived with disabilities (YLD), and economic costs were calculated as absolute values for each country in 2023. 12

Table S3: Projected wellbeing burden of warming-related increase in moderate-to-severe obstructive sleep apnea (OSA) prevalence for different countries under different scenarios from the intergovernmental panel on climate change. Disability-adjusted life years (DALY) rates (per 100,000 persons) were calculated for each scenario (SSP126, SSP245, SSP370 and SSP585) and for each country in 2050 and 2100. 14

Table S4: Projected wellbeing burden of warming-related increase in moderate-to-severe obstructive sleep apnea (OSA) prevalence for different countries under different scenarios from the intergovernmental panel on climate change. Absolute years of life lost (YLL) were calculated for each scenario (SSP126, SSP245, SSP370 and SSP585) and for each country in 2050 and 2100.. 16

Table S5: Projected wellbeing burden of warming-related increase in moderate-to-severe obstructive sleep apnea (OSA) prevalence for different countries under different scenarios from the intergovernmental panel on climate change. Absolute number of years lived with disability (YLD) were calculated for each scenario (SSP126, SSP245, SSP370 and SSP585) and for each country in 2050 and 2100. 18

Table S6: Estimated economic cost associated with the wellbeing burden resulting from warming-related increase in moderate-to-severe obstructive sleep apnea (OSA) prevalence for different countries under different climate models from the intergovernmental panel on climate change. Economic cost (in USD millions) was calculated for each scenario (SSP126, SSP245, SSP370 and SSP585) and for each country in 2050 and 2100. 20

Table S7: Projected wellbeing burden of warming-related increase in moderate-to-severe obstructive sleep apnea (OSA) prevalence for different countries under different scenarios from the intergovernmental panel on climate change as well as different modelling assumptions. Disability-adjusted life years (DALY) rates (per 100,000 persons) were calculated for each scenario (SSP245, SSP370) and for each country in 2050 and 2100. Alternative model accounts for population forecast between 2023 and 2100 and assumes a 30% body mass index-related increase in OSA prevalence between 2023 and 2050.. 22

Table S8: Projected wellbeing burden of warming-related increase in moderate-to-severe obstructive sleep apnea (OSA) prevalence for different countries under different scenarios from the intergovernmental panel on climate change as well as different modelling assumptions. Disability-adjusted life years DALY were calculated for each scenario (SSP245, SSP370) and for each country in 2050 and 2100. Alternative model accounts for population forecast between 2023 and 2100 and assumes a 30% body mass index-related increase in OSA prevalence between 2023 and 2050.. 24

Table S9: Projected workplace productivity loss and associated economic cost resulting from warming-related increase in moderate-to-severe obstructive sleep apnea (OSA) prevalence for different countries. 26

Table S10: Estimated economic cost associated with the labor loss resulting from warming-related increase in moderate-to-severe obstructive sleep apnea (OSA) prevalence for different

countries under different climate models from the intergovernmental panel on climate change. Economic cost (in USD millions) was calculated for each scenario (SSP126, SSP245, SSP370 and SSP585) and for each country in 2050 and 2100. 28
Table S11: Estimated economic cost associated with the labor loss resulting from warming-related increase in moderate-to-severe obstructive sleep apnea (OSA) prevalence for different countries under different climate models from the intergovernmental panel on climate change. Economic cost (in USD millions) per 100,000 persons was calculated for each scenario (SSP126, SSP245, SSP370 and SSP585) and for each country in 2050 and 2100. 30
Table S12: CMIP6 global climate projections, model names and institutions used in this study and downloaded from the Climate Data Store (CDS) of the Copernicus Climate Change Service (C3S). 35

List of figures

Figure S1: Flow chart. 4
Figure S2: Cumulative exposure–response between ambient temperature and nightly obstructive sleep apnea (OSA), including subgroup analyses. 7
Figure S3: Cumulative exposure–response between ambient temperature and nightly severe obstructive sleep apnea (OSA), including subgroup analyses. 8
Figure S4: Cumulative exposure–response between different ambient temperature variables and nightly obstructive sleep apnea (OSA). 9
Figure S5: Exposure-response curves between ambient 24h temperature and the risk ratio (RR) (95%CI) for nightly obstructive sleep apnea (OSA) with different adjustments for air pollution. 10
Figure S6: Exposure-response curves between ambient 24h temperature and the risk ratio (RR) (95%CI) for nightly obstructive sleep apnea (OSA) per quartiles of missing data. 11
Figure S7: Lagged effect of exposure to temperature on the risk of nightly obstructive sleep apnea (OSA). 33
Figure S8: Cumulative lagged exposure-response curve between temperature and risk of nightly obstructive sleep apnea (OSA). 34

Supplementary Results

Participants

There were 125,555 users of the under-mattress sensor between 2017 and September 2023. In instances where there were multiple sleep episodes within a 24h period, we assumed that the longest episode was the main sleep period and discarded the other recordings (~5.7% of recordings). Data before January 2020 were removed given that the under-mattress device was validated in 2020[1]. This resulted in ~6% of the dataset removed. Further inclusion criteria were at least 28 nights of data and an average use of the device of at least 4 times per week, as done in a previous study[2], and having available and valid apnea-hypopnea index (AHI) nightly data.

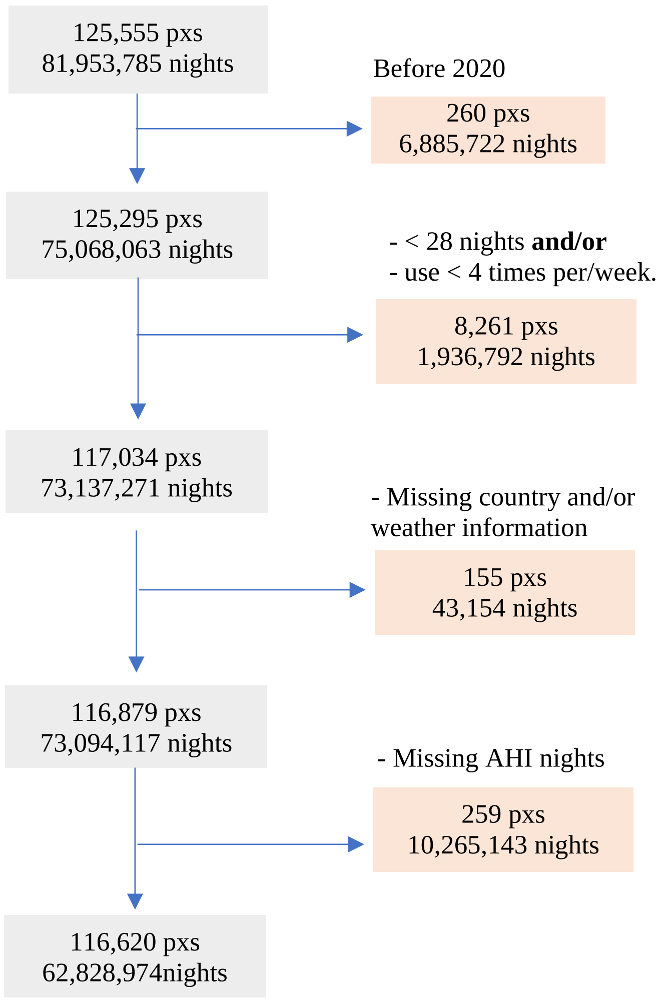

Figure S1: *Flow chart.*

Supplementary Tables and Figures

Table S1: *Risk ratio (OR) (95%CI) for nightly obstructive sleep apnea (OSA) and nightly severe OSA at 99th versus 25th percentile of temperature in each location. Demographics, number of participants, and average number of recordings per participant are included for each location.*

Country	City	Number of participants	Age	Body mass index	Sex (Male) %	Recordings per participant	Moderate-to-severe OSA RR T99	Severe OSA RR at T99
**Australia**	Sydney	1114	45 (14)	29 (7)	77%	359	1.35 (1.17, 1.55)	1.58 (1.19, 2.08)
	Melbourne	825					1.42 (1.18, 1.68)	1.34 (0.99, 1.79)
	Brisbane	483					1.54 (1.30, 1.80)	1.32 (0.94, 1.83)
	Adelaide	375					1.93 (1.53, 2.35)	2.23 (1.15, 3.97)
	Perth	358					1.11 (0.85, 1.39)	0.69 (0.43, 1.09)
**Austria****	Vienna	1345	49 (13)	28 (5)	81%	567	1.02 (0.94, 1.10)	1.15 (1.00, 1.33)
**Belgium**	Brussels	1621	48 (13)	27 (5)	79%	584	1.58 (1.47, 1.70)	1.77 (1.52, 2.05)
**Brazil****	Sao Paulo	188	47 (12)	28 (5)	81%	484	0.93 (0.68, 1.23)	1.10 (0.69, 1.71)
**Bulgaria**	Sofia	112	47 (10)	28 (6)	78%	521	1.80 (1.33, 2.26)	1.15 (0.63, 1.98)
**Canada**	Toronto	1941	47 (14)	28 (6)	75%	526	1.21 (1.11, 1.31)	1.23 (1.06, 1.43)
	Vancouver	504					1.47 (1.21, 1.77)	1.40 (0.96, 2.01)
	Edmonton	291					1.47 (1.24, 1.71)	1.63 (1.21, 2.13)
**China****	Shanghai	296	43 (12)	25 (4)	80%	479	1.10 (0.77, 1.53)	0.67 (0.36, 1.23)
**Croatia****	Zagreb	128	46 (13)	27 (5)	84%	534	1.12 (0.63, 1.80)	1.87 (0.81, 3.93)
**Czech Republic**	Prague	502	44 (12)	28 (5)	78%	492	1.85 (1.59, 2.12)	2.22 (1.65, 2.95)
**Denmark**	Copenhagen	1069	48 (12)	28 (5)	81%	537	1.71 (1.55, 1.88)	1.45 (1.20, 1.75)
**Estonia**	Tallinn	155	45 (11)	27 (5)	74%	556	1.73 (1.20, 2.36)	3.92 (2.20, 6.26)
**Finland**	Helsinki	1932	47 (13)	28 (5)	79%	551	1.70 (1.57, 1.84)	1.75 (1.48, 2.06)
**France**	Paris	17368	50 (14)	27 (5)	75%	540	1.57 (1.54, 1.60)	1.65 (1.57, 1.73)
**Germany**	Berlin	20551	52 (13)	28 (5)	79%	588	1.56 (1.54, 1.59)	1.60 (1.55, 1.65)
**Greece**	Athens	281	48 (12)	28 (5)	81%	505	1.78 (1.29, 2.32)	2.97 (1.48, 5.26)
**Hungary**	Budapest	435	46 (11)	28 (5)	81%	565	1.64 (1.41, 1.88)	1.58 (1.16, 2.12)
**India****	Kolkata	103	49 (16)	26 (5)	80%	396	0.76 (0.46, 1.20)	0.92 (0.41, 1.97)
**Ireland**	Dublin	404	48 (13)	28 (6)	83%	538	1.46 (1.25, 1.67)	1.17 (0.79, 1.71)
**Israel****	Jerusalem	116	48 (13)	28 (5)	80%	540	0.90 (0.56, 1.36)	0.91 (0.42, 1.86)

**Italy**	Rome	1929	51 (13)	27 (5)	85%	545	1.39 (1.28, 1.51)	1.69 (1.44, 1.97)
**Japan**	Tokyo	4764	46 (13)	25 (4)	87%	553	1.95 (1.81, 2.10)	1.60 (1.37, 1.87)
**South Korea****	Seoul	321	41 (12)	25 (4)	80%	459	1.11 (0.75, 1.61)	1.08 (0.54, 2.10)
**Luxembourg**	Luxembourg	263	50 (12)	27 (5)	78%	568	1.72 (1.41, 2.05)	1.43 (0.98, 2.05)
**Mexico**	Mexico City	281	48 (13)	26 (5)	77%	492	1.42 (1.11, 1.74)	1.34 (0.72, 2.30)
**Netherlands**	Amsterdam	2876	48 (13)	27 (5)	77%	552	1.86 (1.74, 1.99)	2.00 (1.75, 2.28)
**New Zealand****	Auckland	155	46 (13)	28 (5)	80%	508	1.22 (0.86, 1.65)	1.86 (0.93, 3.42)
**Norway**	Oslo	976	47 (13)	27 (5)	82%	492	1.56 (1.39, 1.73)	1.33 (1.04, 1.68)
**Poland**	Warsaw	836	46 (12)	27 (5)	81%	478	1.65 (1.49, 1.83)	2.00 (1.59, 2.48)
**Portugal**	Lisbon	586	51 (13)	26 (5)	76%	538	1.43 (1.25, 1.62)	1.29 (0.96, 1.72)
**Romania**	Bucharest	413	45 (12)	28 (5)	79%	418	1.39 (1.14, 1.65)	1.64 (1.14, 2.32)
**Russia**	Moscow	170	46 (12)	27 (5)	78%	450	1.53 (1.09, 2.07)	1.03 (0.50, 2.08)
**Singapore****	Singapore	305	43 (11)	26 (5)	85%	468	1.04 (0.86, 1.25)	0.85 (0.63, 1.13)
**Slovakia**	Bratislava	188	45 (12)	27 (6)	80%	580	1.52 (1.17, 1.93)	1.10 (0.63, 1.90)
**Spain**	Madrid	1569	49 (13)	27 (5)	80%	479	1.29 (1.16, 1.42)	1.27 (1.05, 1.53)
**Sweden**	Stockholm	1838	49 (13)	27 (5)	80%	551	1.57 (1.45, 1.70)	1.71 (1.45, 2.01)
**Switzerland**	Zurich	4553	49 (13)	27 (5)	80%	566	1.79 (1.72, 1.85)	2.08 (1.91, 2.25)
**Thailand****	Bangkok	221	48 (14)	26 (6)	81%	483	1.09 (0.80, 1.45)	0.51 (0.28, 0.94)
**Turkey****	Istanbul	106	49 (13)	27 (4)	76%	436	0.99 (0.54, 1.55)	2.42 (0.98, 4.69)
**United Arab Emirates****	Dubai	134	45 (10)	27 (5)	85%	431	0.97 (0.52, 1.69)	0.31 (0.09, 1.06)
**United Kingdom**	London	7216	49 (13)	28 (6)	79%	567	1.45 (1.40, 1.51)	1.64 (1.54, 1.76)
**United States**	New York	12824	48 (14)	28 (6)	73%	525	1.07 (1.03, 1.11)	1.07 (1.00, 1.14)
	Los Angeles	8083					1.15 (1.10, 1.20)	1.11 (1.03, 1.20)
	Chicago	7345					1.12 (1.08, 1.17)	1.19 (1.11, 1.27)
	Denver	1653					1.15 (1.06, 1.25)	1.13 (0.98, 1.29)
	Detroit	763					1.25 (1.11, 1.40)	1.19 (0.97, 1.45)
	Phoenix	646					1.25 (1.08, 1.42)	1.34 (1.04, 1.71)
	Indiana	387					1.16 (0.98, 1.36)	0.92 (0.67, 1.26)
	Honolulu	122					1.17 (0.96, 1.38)	0.93 (0.62, 1.36)

** countries where we did not find an association between ambient temperatures and OSA prevalence

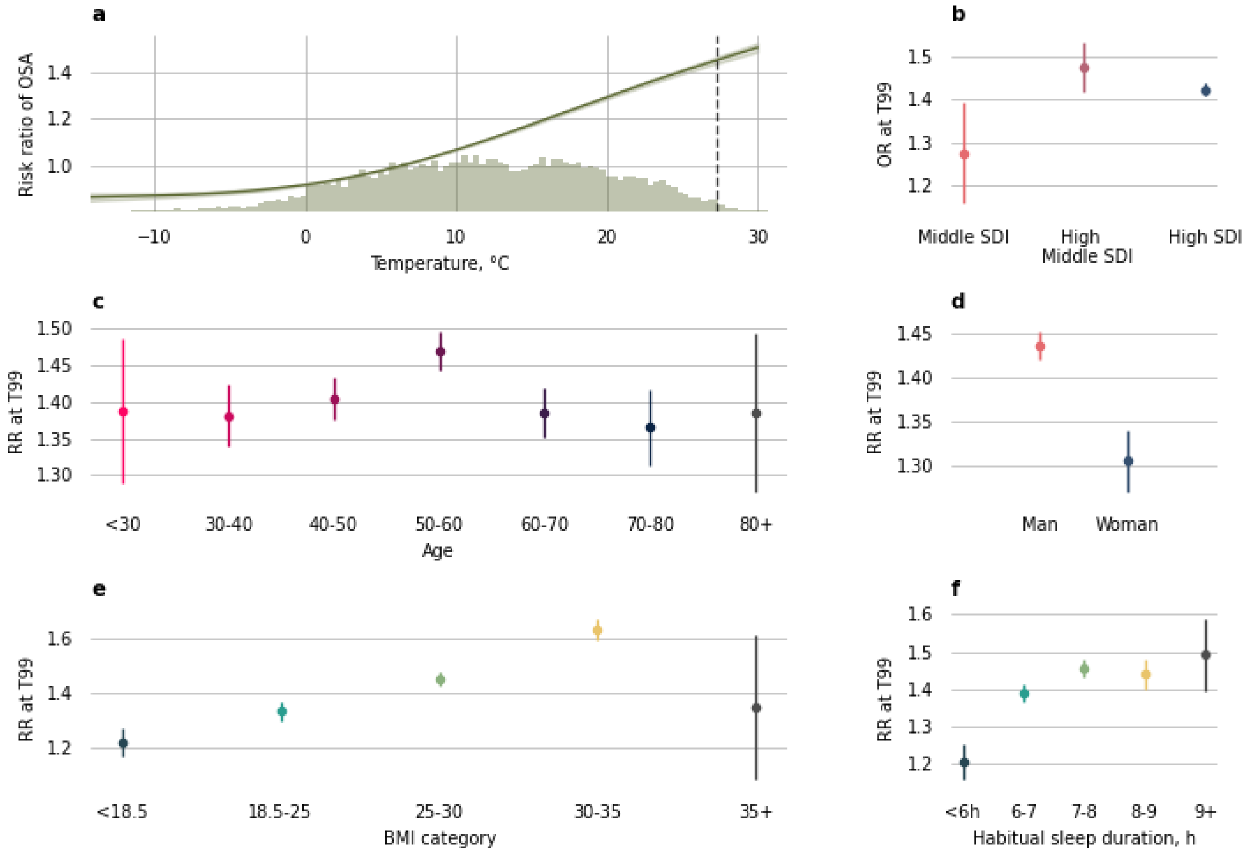

***Figure S2**: Cumulative exposure–response between ambient temperature and nightly obstructive sleep apnea (OSA), including subgroup analyses.*

***a)** Exposure-response curve between ambient temperature and the risk ratio (RR) (95%CI) for nightly obstructive sleep apnea (OSA). Distribution of temperature (histogram) and temperature at the 99th percentile (dashed black line) are also highlighted. **b-f)** Subgroup analyses including the RR (95%CI) for nightly OSA at 99th vs. 25th percentile of temperature based on: **b)** country of residence's socio-developmental index (SDI) categories, **c)** age categories, **d)** sex, **e)** body mass index (BMI) categories, and **f)** habitual sleep duration categories.*

Sample size N. BMI (note ~15% of the sample is missing weight measurements): <15.8: 1239, 18.5 to 25: 31012, 25 to 30: 34206, 30 to 35: 15832, >35: 8627. Age: <30: 9366, 30 to 40: 25273, 40 to 50: 30598, 50 to 60: 28253, 60 to 70: 15368, 70 to 80: 6162, >80: 1590; Sex: Male: 90131, Female: 26479; SDI category: Middle SDI: 1816, High-Middle SDI: 7136, High SDI: 106959. Habitual sleep duration (N represents person-years), <6 hours: 21270, 6 to 7:52571, 7 to 8: 64584, 8 to 9: 24407, >9: 5074.

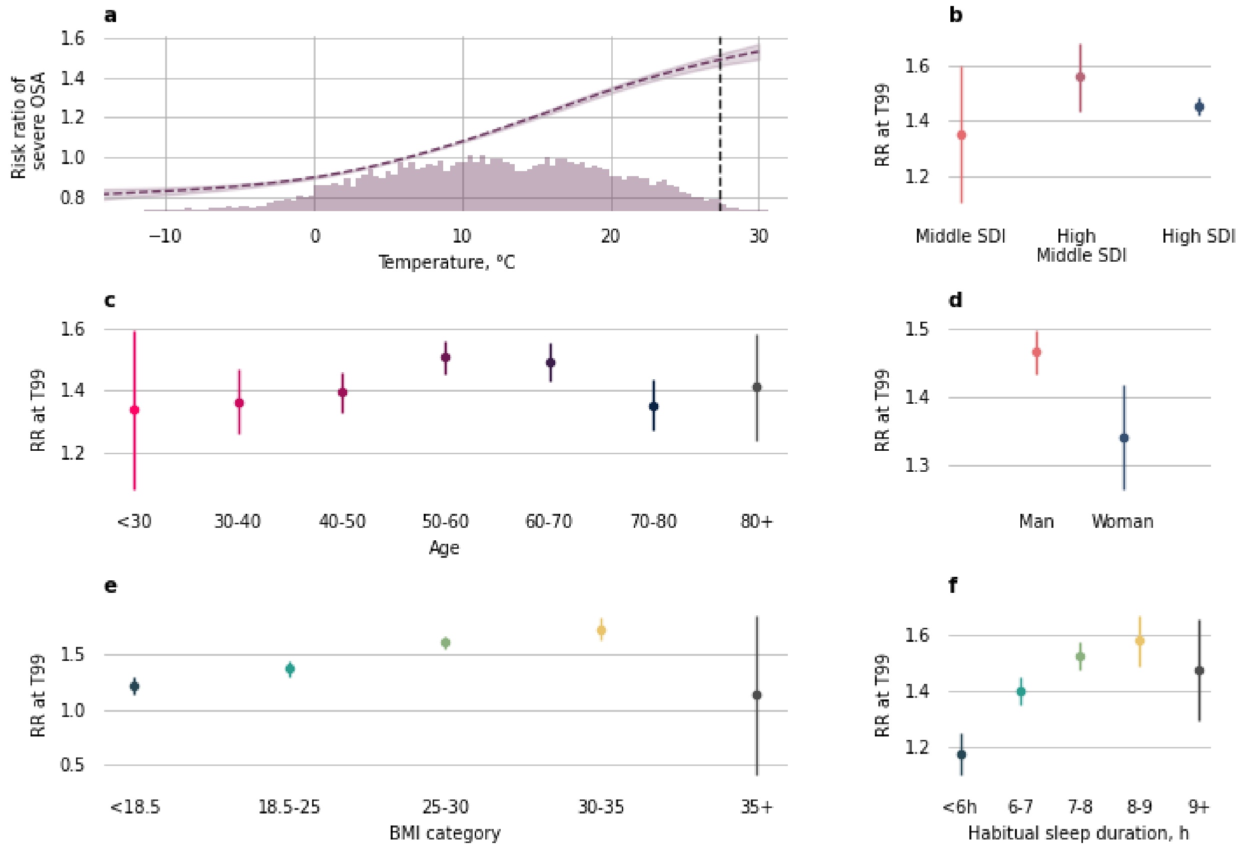

Figure S3: *Cumulative exposure–response between ambient temperature and nightly severe obstructive sleep apnea (OSA), including subgroup analyses.*

a) *Exposure-response curve between ambient temperature and the risk ratio (RR) (95%CI) for nightly severe obstructive sleep apnea (OSA). Distribution of temperature (histogram) and temperature at the 99th percentile (dashed black line) are also highlighted.* ***b-f)*** *Subgroup analyses including the RR (95%CI) for nightly severe OSA at 99th vs. 25th percentile of temperature based on:* ***b)*** *country of residence socio-developmental index (SDI) categories,* ***c)*** *age categories,* ***d)*** *sex,* ***e)*** *body mass index (BMI) categories, and* ***f)*** *habitual sleep duration categories.*

Sample size N. BMI (note ~15% of the sample is missing weight measurements): <15.8: 1239, 18.5 to 25: 31012, 25 to 30: 34206, 30 to 35: 15832, >35: 8627; Age: <30: 9366, 30 to 40: 25273, 40 to 50: 30598, 50 to 60: 28253, 60 to 70: 15368, 70 to 80: 6162, >80: 1590; Sex, Male: 90131 , Female: 26479; SDI category: Middle SDI: 1816, High-Middle SDI: 7136, High SDI: 106959. Habitual sleep duration (N represents person-years), <6 hours: 21270, 6 to 7:52571, 7 to 8: 64584, 8 to 9:24407, >9: 5074.

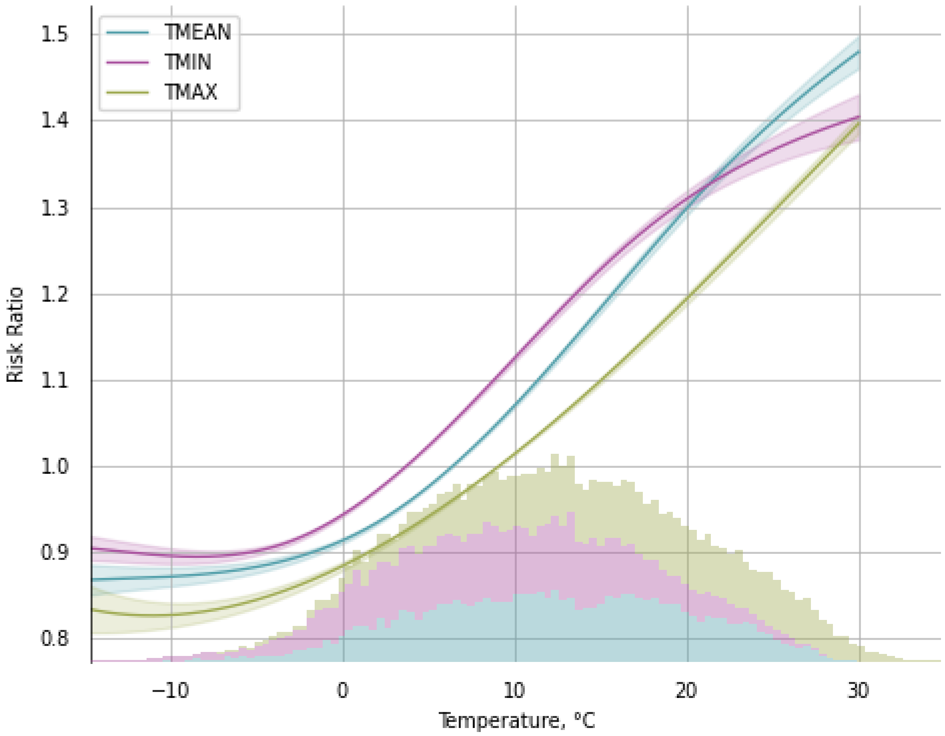

Figure S4: *Cumulative exposure–response between different ambient temperature variables and nightly obstructive sleep apnea (OSA).*

Exposure-response curves between ambient mean (blue), maximum, (green) and minimum (purple) 24h temperature and the risk ratio (RR) (95%CI) for nightly obstructive sleep apnea (OSA). Reference values for each exposure-response curve were set to the 25th percentile.

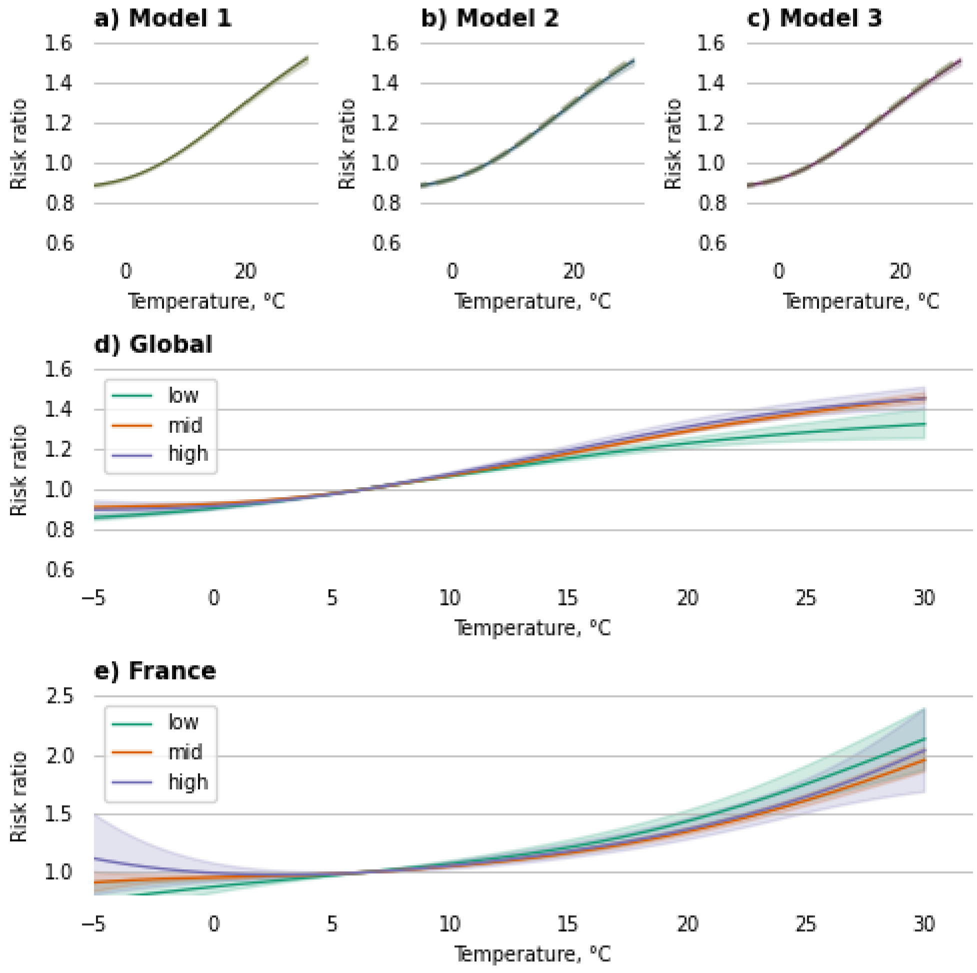

Figure S5: *Exposure-response curves between ambient 24h temperature and the risk ratio (RR) (95%CI) for nightly obstructive sleep apnea (OSA) with different adjustments for air pollution.*

a) *Model 1 was adjusted for all confounders (see methods section) except air pollution.* ***b)*** *Model 2 and* ***c)*** *Model 3 were additionally adjusted for particulate matter with aerodynamic diameter <2.5 and 10µm, respectively.* ***d****) Exposure-response (global) curve between temperature and risk ratio of nightly OSA at different levels (low: below 15th percentile, mid :15th to 85th percentiles, high: above 85th percentile).* ***e****) Same as d) but for single country (France).*

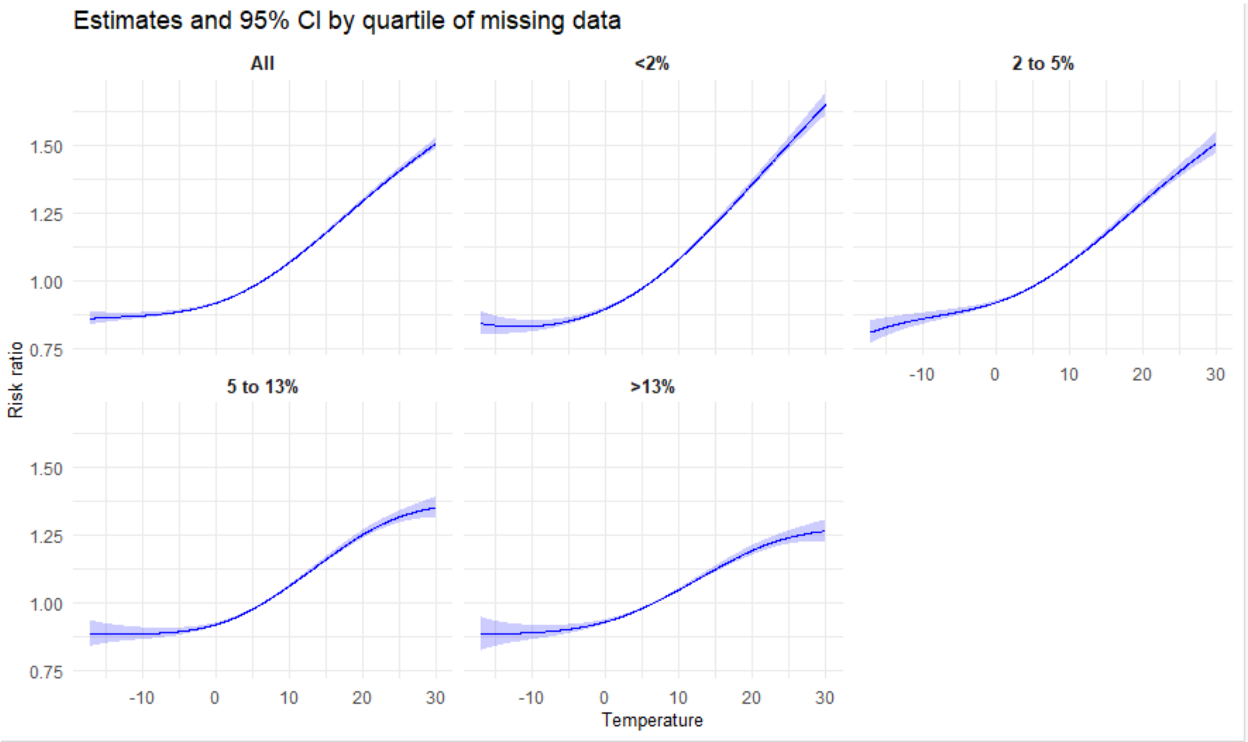

Figure S6: *Exposure-response curves between ambient 24h temperature and the risk ratio (RR) (95%CI) for nightly obstructive sleep apnea (OSA) per quartiles of missing data.*

Note that lower amount of missing data (<2%) had a higher effect size in the association between nightly OSA and temperature vs higher amount of missing data. Missing data was calculated as the proportion of night missing per participants due to sleep duration less than 5 hours.

Table S2: *Projected wellbeing burden and associated cost of warming-related increase in moderate-to-severe obstructive sleep apnea (OSA) prevalence for different countries. Disability-adjusted life years (DALY) rates were calculated for each country in 2000 and 2023, and the % change was calculated. Years of life lost (YLL), years lived with disabilities (YLD), and economic costs were calculated as absolute values for each country in 2023.*

Country	DALY rates per 100,000		% change	DALY	YLL	YLD	Cost (millions)	Population (millions)
	2000	2023		2023	2023	2023	2023	2023
**total**	47 (29, 65)	100 (62, 137)	112%	788198 (489226, 1087170)	27180 (13590, 40770)	761018 (475636, 1046400)	68327 (34163, 102490)	1022.2
**Australia#**	95 (59, 131)	262 (163, 362)	175%	52719 (32750, 72689)	1597 (798, 2395)	51122 (31951, 70293)	6823 (3412, 10235)	20.1
**Belgium**	62 (38, 85)	125 (78, 173)	102%	11442 (7109, 15775)	334 (167, 501)	11108 (6942, 15273)	1224 (612, 1836)	9.1
**Bulgaria**	75 (46, 103)	118 (73, 162)	57%	6442 (3994, 8891)	264 (132, 396)	6179 (3862, 8496)	204 (102, 305)	5.5
**Canada#**	9 (6, 13)	17 (10, 23)	88%	5222 (3242, 7201)	173 (86, 259)	5049 (3155, 6942)	557 (279, 836)	31.1
**Czech Republic**	46 (29, 63)	96 (60, 132)	109%	8212 (5102, 11321)	243 (121, 364)	7969 (4981, 10957)	500 (250, 750)	8.6
**Denmark**	68 (42, 93)	121 (75, 166)	78%	5618 (3498, 7738)	102 (51, 153)	5516 (3447, 7584)	764 (382, 1146)	4.7
**Estonia**	35 (22, 49)	74 (46, 102)	111%	796 (495, 1097)	22 (11, 34)	774 (484, 1064)	47 (24, 71)	1.1
**Finland**	33 (21, 46)	73 (46, 101)	121%	3270 (2036, 4504)	61 (30, 91)	3209 (2006, 4412)	352 (176, 527)	4.5
**France**	56 (35, 77)	134 (83, 184)	139%	68336 (42479, 94193)	1848 (924, 2772)	66488 (41555, 91421)	6077 (3038, 9115)	51.2
**Germany**	59 (36, 81)	116 (72, 160)	97%	80266 (49964, 110567)	1618 (809, 2427)	78648 (49155, 108141)	8467 (4234, 12701)	68.9
**Greece**	57 (35, 79)	106 (66, 146)	86%	8826 (5467, 12186)	397 (198, 595)	8429 (5268, 11590)	406 (203, 609)	8.3
**Hungary**	40 (25, 55)	96 (59, 132)	140%	7442 (4625, 10259)	209 (105, 314)	7233 (4520, 9945)	330 (165, 494)	7.8
**Ireland**	34 (21, 47)	70 (44, 97)	106%	2710 (1688, 3732)	46 (23, 69)	2664 (1665, 3663)	562 (281, 843)	3.9
**Italy**	36 (22, 50)	97 (60, 134)	169%	47854 (29785, 65924)	993 (497, 1490)	46861 (29288, 64434)	3673 (1836, 5509)	49.4

**Japan**	39<br>(25, 54)	120<br>(75, 165)	207%	124773<br>(77778, 171767)	1640<br>(820, 2460)	123133<br>(76958, 169308)	8443<br>(4222, 12665)	104.2
**Luxembourg**	75<br>(47, 104)	151<br>(94, 208)	101%	793<br>(493, 1093)	20<br>(10, 30)	773<br>(483, 1062)	203<br>(102, 305)	0.5
**Mexico**	76<br>(47, 106)	112<br>(69, 156)	47%	96844<br>(59460, 134229)	8545<br>(4273, 12818)	88299<br>(55187, 121411)	2697<br>(1349, 4046)	86.3
**Netherlands**	80<br>(50, 111)	158<br>(98, 217)	97%	22611<br>(14091, 31132)	331<br>(165, 496)	22281<br>(13925, 30636)	2828<br>(1414, 4242)	14.3
**Norway**	45<br>(28, 62)	71<br>(44, 97)	58%	3020<br>(1881, 4158)	49<br>(24, 73)	2971<br>(1857, 4085)	531<br>(266, 797)	4.3
**Poland**	55<br>(34, 76)	103<br>(64, 142)	87%	31967<br>(19811, 44123)	1349<br>(674, 2023)	30618<br>(19136, 42100)	1414<br>(707, 2121)	31.0
**Portugal**	37<br>(23, 51)	87<br>(54, 120)	135%	7462<br>(4640, 10285)	195<br>(97, 292)	7268<br>(4542, 9993)	407<br>(204, 611)	8.6
**Romania**	21<br>(13, 29)	74<br>(46, 102)	252%	11127<br>(6893, 15361)	490<br>(245, 734)	10637<br>(6648, 14626)	410<br>(205, 615)	15.0
**Russia**	-5<br>(-3, -7)	10<br>(6, 14)	n.a.	11713<br>(7205, 16220)	920<br>(460, 1380)	10793<br>(6745, 14840)	324<br>(162, 486)	112.2
**Slovakia**	30<br>(19, 42)	48<br>(30, 67)	60%	2106<br>(1307, 2905)	73<br>(36, 109)	2033<br>(1271, 2795)	103<br>(52, 155)	4.4
**Spain**	37<br>(23, 51)	63<br>(39, 87)	70%	24662<br>(15357, 33967)	454<br>(227, 681)	24208<br>(15130, 33286)	1612<br>(806, 2418)	39.0
**Sweden**	46<br>(29, 64)	86<br>(54, 119)	87%	6993<br>(4359, 9628)	97<br>(49, 146)	6896<br>(4310, 9482)	788<br>(394, 1181)	8.1
**Switzerland**	66<br>(41, 91)	164<br>(102, 226)	148%	11679<br>(7280, 16078)	157<br>(79, 236)	11522<br>(7201, 15842)	2336<br>(1168, 3504)	7.1
**United Kingdom**	59<br>(37, 81)	113<br>(70, 155)	92%	59365<br>(36975, 81755)	1024<br>(512, 1536)	58341<br>(36463, 80219)	5802<br>(2901, 8703)	52.7
**United States**[#]	9<br>(5, 12)	25<br>(15, 34)	177%	63927<br>(39463, 88391)	3929<br>(1964, 5893)	59998<br>(37499, 82498)	10445<br>(5223, 15668)	260.4

[#]Australia, the US, and Canada estimates were based on locations within the country with the most users (Sydney, New York, and Toronto, respectively). This led to conservative estimates given the most populated locations in these 3 countries had a smaller effect size on the association between ambient temperature and OSA (See Table S1)

Table S3: *Projected wellbeing burden of warming-related increase in moderate-to-severe obstructive sleep apnea (OSA) prevalence for different countries under different scenarios from the intergovernmental panel on climate change. Disability-adjusted life years (DALY) rates (per 100,000 persons) were calculated for each scenario (SSP126, SSP245, SSP370 and SSP585) and for each country in 2050 and 2100.*

	2050				2100			
**country**	**SSP126**	**SSP245**	**SSP370**	**SSP585**	**SSP126**	**SSP245**	**SSP370**	**SSP585**
**total**	116<br>(96, 136)	126<br>(101, 151)	133<br>(106, 159)	144<br>(115, 174)	114<br>(95, 133)	162<br>(123, 201)	221<br>(161, 282)	279<br>(198, 360)
**Australia**	273<br>(260, 286)	276<br>(260, 292)	282<br>(265, 299)	285<br>(267, 303)	277<br>(263, 291)	295<br>(272, 317)	329<br>(295, 364)	369<br>(319, 420)
**Belgium**	142<br>(121, 162)	135<br>(112, 158)	157<br>(131, 184)	161<br>(134, 189)	134<br>(116, 152)	173<br>(136, 210)	236<br>(180, 293)	294<br>(216, 372)
**Bulgaria**	131<br>(102, 160)	188<br>(140, 236)	179<br>(129, 228)	214<br>(157, 270)	137<br>(106, 167)	238<br>(171, 306)	325<br>(220, 431)	436<br>(295, 577)
**Canada**	27<br>(16, 38)	30<br>(18, 42)	32<br>(19, 45)	38<br>(24, 53)	27<br>(16, 37)	46<br>(28, 64)	59<br>(36, 82)	76<br>(47, 104)
**Czech Republic**	112<br>(105, 119)	109<br>(99, 119)	122<br>(110, 134)	130<br>(115, 144)	110<br>(103, 116)	137<br>(117, 157)	193<br>(154, 233)	278<br>(208, 348)
**Denmark**	144<br>(118, 171)	147<br>(114, 180)	160<br>(124, 196)	179<br>(140, 217)	141<br>(116, 166)	200<br>(147, 253)	280<br>(198, 361)	363<br>(255, 472)
**Estonia**	77<br>(67, 86)	94<br>(78, 109)	91<br>(76, 106)	95<br>(79, 111)	83<br>(71, 95)	112<br>(89, 134)	140<br>(106, 174)	173<br>(127, 218)
**Finland**	89<br>(84, 93)	99<br>(88, 110)	96<br>(88, 105)	117<br>(103, 132)	97<br>(90, 105)	136<br>(111, 161)	186<br>(144, 229)	237<br>(177, 296)
**France**	152<br>(136, 168)	147<br>(128, 166)	174<br>(151, 197)	168<br>(145, 190)	149<br>(134, 164)	181<br>(150, 213)	263<br>(206, 320)	313<br>(235, 391)
**Germany**	137<br>(113, 160)	130<br>(104, 156)	152<br>(121, 182)	164<br>(130, 197)	132<br>(111, 154)	180<br>(135, 224)	243<br>(178, 307)	317<br>(226, 407)
**Greece**	129<br>(92, 166)	155<br>(106, 203)	169<br>(117, 220)	189<br>(131, 247)	128<br>(91, 165)	209<br>(140, 278)	305<br>(201, 408)	383<br>(251, 516)
**Hungary**	108<br>(80, 137)	134<br>(95, 173)	137<br>(96, 177)	150<br>(106, 194)	106<br>(78, 134)	170<br>(118, 223)	233<br>(155, 310)	310<br>(206, 415)
**Ireland**	85<br>(84, 87)	79<br>(77, 81)	93<br>(90, 95)	95<br>(90, 101)	82<br>(82, 83)	117<br>(101, 134)	174<br>(141, 207)	212<br>(162, 261)
**Italy**	116<br>(82, 150)	140<br>(97, 183)	139<br>(98, 181)	158<br>(111, 205)	115<br>(81, 148)	175<br>(119, 232)	237<br>(158, 316)	296<br>(197, 394)
**Japan**	137<br>(100, 174)	168<br>(124, 213)	160<br>(115, 205)	191<br>(137, 245)	145<br>(105, 186)	214<br>(152, 276)	283<br>(192, 374)	361<br>(244, 479)

**Luxembourg**	173<br>(154, 193)	173<br>(150, 197)	205<br>(176, 234)	201<br>(171, 230)	162<br>(147, 178)	226<br>(182, 269)	320<br>(248, 393)	375<br>(279, 470)
**Mexico**	140<br>(122, 158)	147<br>(123, 170)	167<br>(140, 193)	175<br>(140, 209)	147<br>(126, 167)	206<br>(160, 252)	302<br>(223, 380)	401<br>(279, 523)
**Netherlands**	176<br>(143, 209)	173<br>(137, 210)	197<br>(156, 238)	211<br>(167, 255)	167<br>(138, 197)	229<br>(171, 286)	308<br>(225, 391)	379<br>(271, 486)
**Norway**	85<br>(70, 99)	96<br>(75, 117)	100<br>(79, 122)	117<br>(93, 142)	88<br>(72, 104)	138<br>(101, 174)	190<br>(135, 246)	243<br>(171, 314)
**Poland**	118<br>(97, 139)	122<br>(97, 147)	132<br>(103, 161)	145<br>(115, 175)	113<br>(94, 132)	165<br>(123, 206)	206<br>(149, 263)	290<br>(205, 376)
**Portugal**	113<br>(86, 141)	119<br>(89, 150)	124<br>(93, 155)	132<br>(98, 166)	99<br>(78, 121)	156<br>(112, 200)	233<br>(160, 306)	279<br>(189, 369)
**Romania**	82<br>(57, 107)	108<br>(74, 142)	100<br>(66, 134)	111<br>(75, 146)	81<br>(56, 105)	127<br>(86, 169)	159<br>(102, 215)	202<br>(132, 273)
**Russia**	29<br>(29, 29)	31<br>(28, 33)	17<br>(20, 15)	37<br>(32, 43)	27<br>(27, 26)	50<br>(40, 59)	79<br>(58, 101)	101<br>(71, 131)
**Slovakia**	58<br>(56, 60)	69<br>(62, 76)	74<br>(65, 84)	78<br>(68, 88)	58<br>(56, 59)	89<br>(74, 103)	131<br>(100, 162)	183<br>(134, 233)
**Spain**	86<br>(62, 111)	95<br>(66, 123)	90<br>(63, 117)	103<br>(73, 134)	74<br>(54, 95)	123<br>(84, 163)	162<br>(108, 216)	186<br>(124, 247)
**Sweden**	100<br>(77, 123)	123<br>(90, 156)	117<br>(84, 150)	133<br>(97, 170)	105<br>(80, 130)	161<br>(114, 208)	214<br>(144, 284)	271<br>(183, 360)
**Switzerland**	189<br>(160, 218)	197<br>(162, 232)	211<br>(174, 248)	222<br>(182, 263)	186<br>(158, 214)	239<br>(188, 290)	340<br>(254, 426)	433<br>(313, 553)
**United Kingdom**	128<br>(107, 149)	126<br>(104, 149)	146<br>(120, 171)	149<br>(121, 176)	113<br>(97, 129)	167<br>(129, 204)	237<br>(178, 297)	277<br>(201, 353)
**United States**	33<br>(19, 46)	38<br>(23, 53)	33<br>(19, 47)	39<br>(22, 56)	33<br>(19, 48)	48<br>(29, 66)	54<br>(32, 76)	59<br>(34, 83)

Table S4: *Projected wellbeing burden of warming-related increase in moderate-to-severe obstructive sleep apnea (OSA) prevalence for different countries under different scenarios from the intergovernmental panel on climate change. Absolute years of life lost (YLL) were calculated for each scenario (SSP126, SSP245, SSP370 and SSP585) and for each country in 2050 and 2100.*

	2050				2100			
**country**	**SSP126**	**SSP245**	**SSP370**	**SSP585**	**SSP126**	**SSP245**	**SSP370**	**SSP585**
**total**	34342 (25730, 42954)	37045 (26601, 47488)	37655 (27243, 48068)	42424 (29228, 55620)	34486 (25802, 43170)	49473 (32815, 66130)	67737 (42284, 93189)	85439 (50736, 120142)
**Australia**	1662 (1559, 1765)	1680 (1555, 1806)	1718 (1583, 1853)	1734 (1589, 1880)	1686 (1571, 1802)	1794 (1611, 1976)	2006 (1727, 2285)	2250 (1846, 2653)
**Belgium**	378 (305, 451)	360 (280, 441)	420 (327, 513)	431 (334, 528)	359 (295, 422)	462 (331, 592)	631 (432, 830)	785 (511, 1058)
**Bulgaria**	294 (209, 379)	421 (279, 562)	400 (254, 547)	479 (313, 645)	306 (215, 397)	535 (336, 733)	730 (418, 1041)	977 (562, 1392)
**Canada**	279 (134, 425)	311 (152, 470)	327 (151, 502)	396 (202, 589)	276 (132, 420)	477 (235, 719)	609 (293, 926)	782 (395, 1168)
**Czech Republic**	283 (259, 306)	276 (243, 308)	308 (267, 349)	328 (280, 375)	277 (256, 298)	346 (278, 414)	488 (357, 620)	702 (468, 937)
**Denmark**	122 (92, 152)	125 (88, 162)	135 (95, 176)	151 (108, 194)	119 (91, 148)	169 (110, 229)	236 (146, 327)	307 (186, 428)
**Estonia**	23 (19, 27)	28 (22, 34)	27 (21, 33)	29 (22, 35)	25 (20, 30)	34 (25, 43)	42 (29, 56)	52 (34, 70)
**Finland**	74 (69, 78)	82 (70, 94)	80 (71, 89)	97 (81, 113)	81 (72, 89)	113 (85, 140)	154 (108, 201)	196 (131, 261)
**France**	2103 (1810, 2395)	2039 (1693, 2385)	2404 (1983, 2824)	2321 (1906, 2737)	2062 (1790, 2335)	2510 (1929, 3091)	3642 (2602, 4682)	4334 (2912, 5755)
**Germany**	1900 (1470, 2330)	1805 (1326, 2283)	2108 (1546, 2670)	2272 (1663, 2881)	1839 (1440, 2238)	2495 (1671, 3318)	3370 (2178, 4563)	4398 (2726, 6071)
**Greece**	483 (300, 666)	579 (341, 816)	632 (379, 884)	706 (420, 993)	479 (298, 660)	781 (442, 1121)	1140 (633, 1647)	1435 (785, 2085)
**Hungary**	238 (155, 320)	294 (181, 407)	299 (181, 418)	329 (202, 457)	232 (152, 313)	373 (220, 525)	509 (286, 733)	680 (378, 982)
**Ireland**	56 (54, 58)	52 (50, 54)	61 (59, 63)	63 (58, 67)	54 (53, 55)	77 (62, 91)	114 (85, 143)	139 (96, 182)
**Italy**	1189 (729, 1649)	1435 (848, 2023)	1429 (861, 1996)	1621 (987, 2254)	1177 (723, 1631)	1797 (1029, 2566)	2429 (1362, 3497)	3030 (1692, 4369)
**Japan**	1878 (1201, 2555)	2302 (1492, 3112)	2190 (1377, 3002)	2616 (1642, 3589)	1989 (1257, 2722)	2932 (1807, 4058)	3874 (2219, 5528)	4947 (2808, 7086)
**Luxembourg**	23 (20, 27)	23 (19, 27)	27 (22, 32)	27 (22, 32)	22 (19, 24)	30 (22, 38)	43 (30, 56)	50 (33, 67)
**Mexico**	10655 (8875, 12436)	11157 (8868, 13445)	12703 (10083, 15324)	13282 (9870, 16693)	11173 (9134, 13212)	15683 (11131, 20235)	22948 (15205, 30690)	30531 (18495, 42568)

**Netherlands**	369<br>(278, 461)	363<br>(261, 465)	413<br>(300, 527)	442<br>(319, 565)	351<br>(269, 433)	480<br>(320, 640)	646<br>(416, 876)	794<br>(495, 1093)
**Norway**	58<br>(45, 72)	66<br>(47, 85)	69<br>(50, 89)	81<br>(58, 103)	61<br>(46, 75)	95<br>(62, 128)	131<br>(81, 182)	167<br>(102, 233)
**Poland**	1540<br>(1178, 1902)	1590<br>(1160, 2020)	1730<br>(1232, 2227)	1895<br>(1378, 2413)	1479<br>(1147, 1810)	2153<br>(1442, 2865)	2689<br>(1711, 3666)	3793<br>(2327, 5259)
**Portugal**	254<br>(173, 334)	267<br>(177, 357)	277<br>(185, 370)	295<br>(195, 396)	222<br>(158, 287)	349<br>(218, 479)	521<br>(307, 736)	624<br>(359, 890)
**Romania**	543<br>(325, 760)	711<br>(415, 1007)	658<br>(363, 953)	732<br>(424, 1040)	532<br>(320, 744)	840<br>(479, 1200)	1049<br>(558, 1539)	1336<br>(726, 1946)
**Russia**	2534<br>(2508, 2560)	2696<br>(2436, 2955)	1519<br>(1803, 1235)	3306<br>(2706, 3905)	2375<br>(2428, 2322)	4375<br>(3276, 5474)	7000<br>(4543, 9457)	8907<br>(5507, 12307)
**Slovakia**	88<br>(84, 91)	104<br>(90, 118)	112<br>(94, 131)	118<br>(99, 138)	87<br>(84, 91)	134<br>(105, 163)	198<br>(137, 259)	277<br>(178, 376)
**Spain**	621<br>(385, 858)	680<br>(409, 952)	646<br>(391, 901)	742<br>(453, 1032)	535<br>(342, 729)	886<br>(512, 1261)	1163<br>(649, 1677)	1336<br>(749, 1922)
**Sweden**	113<br>(78, 148)	139<br>(90, 189)	132<br>(82, 182)	150<br>(96, 205)	118<br>(80, 156)	182<br>(111, 252)	242<br>(137, 347)	306<br>(174, 438)
**Switzerland**	181<br>(145, 218)	188<br>(144, 233)	202<br>(155, 249)	213<br>(161, 264)	178<br>(143, 213)	229<br>(164, 294)	326<br>(217, 435)	414<br>(262, 567)
**United Kingdom**	1166<br>(909, 1423)	1151<br>(879, 1422)	1324<br>(1022, 1627)	1353<br>(1021, 1685)	1029<br>(840, 1217)	1517<br>(1062, 1971)	2160<br>(1439, 2880)	2519<br>(1604, 3435)
**United States**	5237<br>(2362, 8111)	6121<br>(2986, 9256)	5305<br>(2308, 8301)	6215<br>(2618, 9811)	5362<br>(2425, 8299)	7627<br>(3739, 11515)	8644<br>(3978, 13310)	9369<br>(4195, 14542)

Table S5: *Projected wellbeing burden of warming-related increase in moderate-to-severe obstructive sleep apnea (OSA) prevalence for different countries under different scenarios from the intergovernmental panel on climate change. Absolute number of years lived with disability (YLD) were calculated for each scenario (SSP126, SSP245, SSP370 and SSP585) and for each country in 2050 and 2100.*

	2050				2100			
**country**	**SSP126**	**SSP245**	**SSP370**	**SSP585**	**SSP126**	**SSP245**	**SSP370**	**SSP585**
**total**	916798 (734032, 1099564)	992573 (774113, 1211032)	1022811 (799260, 1246361)	1137318 (872975, 1401661)	909413 (729416, 1089409)	1293592 (962250, 1624934)	1746574 (1251612, 2241536)	2183438 (1526800, 2840076)
**Australia**	53197 (50723, 55672)	53792 (50771, 56812)	54990 (51750, 58230)	55523 (52021, 59024)	53981 (51213, 56749)	57420 (53039, 61801)	64223 (57520, 70925)	72022 (62333, 81711)
**Belgium**	12554 (10741, 14367)	11977 (9979, 13975)	13955 (11635, 16275)	14322 (11915, 16729)	11919 (10344, 13493)	15336 (12078, 18594)	20975 (16022, 25927)	26079 (19264, 32895)
**Bulgaria**	6879 (5387, 8371)	9853 (7371, 12334)	9377 (6798, 11956)	11222 (8307, 14136)	7168 (5568, 8768)	12520 (9038, 16002)	17083 (11614, 22552)	22886 (15597, 30174)
**Canada**	8150 (4968, 11331)	9065 (5585, 12545)	9534 (5699, 13369)	11548 (7316, 15779)	8065 (4915, 11214)	13922 (8620, 19223)	17790 (10858, 24721)	22813 (14357, 31270)
**Czech Republic**	9276 (8689, 9864)	9051 (8247, 9856)	10114 (9099, 11128)	10753 (9588, 11919)	9088 (8571, 9605)	11361 (9690, 13032)	16033 (12799, 19267)	23054 (17276, 28832)
**Denmark**	6598 (5388, 7808)	6740 (5241, 8239)	7320 (5681, 8960)	8178 (6428, 9928)	6448 (5294, 7602)	9153 (6749, 11557)	12785 (9096, 16473)	16613 (11700, 21526)
**Estonia**	801 (705, 897)	980 (820, 1139)	947 (791, 1103)	994 (829, 1160)	867 (746, 987)	1167 (937, 1397)	1467 (1116, 1818)	1806 (1336, 2276)
**Finland**	3883 (3694, 4073)	4333 (3846, 4820)	4216 (3847, 4586)	5127 (4494, 5759)	4250 (3923, 4577)	5942 (4851, 7033)	8142 (6300, 9984)	10339 (7752, 12926)
**France**	75661 (67769, 83554)	73370 (64042, 82698)	86488 (75136, 97841)	83520 (72308, 94732)	74214 (66864, 81564)	90306 (74627, 105985)	131062 (102994, 159129)	155932 (117565, 194298)
**Germany**	92375 (76704, 108045)	87728 (70276, 105181)	102491 (82006, 122975)	110445 (88242, 132649)	89398 (74844, 103952)	121270 (91240, 151300)	163853 (120358, 207349)	213825 (152854, 274796)
**Greece**	10261 (7351, 13170)	12290 (8501, 16079)	13416 (9390, 17442)	15005 (10447, 19564)	10176 (7298, 13053)	16599 (11194, 22003)	24216 (16140, 32292)	30480 (20119, 40841)
**Hungary**	8207 (6061, 10353)	10155 (7228, 13081)	10334 (7266, 13402)	11382 (8089, 14675)	8031 (5951, 10111)	12880 (8931, 16828)	17598 (11806, 23391)	23489 (15656, 31322)
**Ireland**	3243 (3172, 3313)	3009 (2926, 3092)	3515 (3422, 3607)	3618 (3409, 3827)	3128 (3101, 3156)	4446 (3824, 5067)	6607 (5355, 7860)	8047 (6177, 9917)
**Italy**	56113 (39831, 72395)	67729 (46930, 88528)	67417 (47339, 87495)	76486 (54058, 98914)	55537 (39471, 71603)	84824 (57615, 112034)	114642 (76854, 152430)	143011 (95636, 190385)
**Japan**	141035 (102920, 179150)	172874 (127248, 218500)	164426 (118669, 210182)	196440 (141614, 251265)	149387 (108140, 190634)	220202 (156828, 283576)	290888 (197708, 384069)	371499 (251026, 491972)

**Luxembourg**	887 (787, 986)	888 (767, 1009)	1050 (903, 1197)	1027 (877, 1177)	830 (752, 908)	1155 (934, 1376)	1641 (1272, 2010)	1918 (1433, 2402)
**Mexico**	110101 (96305, 123897)	115280 (97543, 133016)	131262 (110955, 151568)	137240 (110802, 163678)	115451 (99649, 131253)	162052 (126776, 197328)	237115 (177114, 297117)	315476 (222200, 408753)
**Netherlands**	24854 (20231, 29476)	24446 (19305, 29588)	27835 (22096, 33573)	29762 (23558, 35966)	23631 (19467, 27796)	32300 (24213, 40387)	43480 (31874, 55086)	53455 (38366, 68544)
**Norway**	3558 (2940, 4175)	4038 (3176, 4900)	4221 (3325, 5118)	4930 (3904, 5955)	3699 (3029, 4369)	5790 (4271, 7308)	8009 (5692, 10326)	10213 (7206, 13220)
**Poland**	34947 (28786, 41109)	36092 (28771, 43412)	39260 (30787, 47734)	43018 (34208, 51828)	33564 (27921, 39207)	48880 (36764, 60996)	61030 (44393, 77667)	86095 (61131, 111058)
**Portugal**	9458 (7209, 11707)	9960 (7450, 12469)	10338 (7747, 12928)	11019 (8196, 13842)	8293 (6481, 10106)	13004 (9353, 16655)	19443 (13438, 25447)	23283 (15861, 30706)
**Romania**	11787 (8250, 15323)	15447 (10620, 20274)	14290 (9487, 19093)	15896 (10876, 20916)	11559 (8108, 15010)	18241 (12366, 24115)	22784 (14796, 30772)	29025 (19082, 38968)
**Russia**	29717 (29488, 29945)	31618 (29336, 33900)	17817 (20311, 15322)	38773 (33501, 44045)	27856 (28325, 27386)	51312 (41645, 60979)	82106 (60492, 103720)	104466 (74559, 134372)
**Slovakia**	2446 (2370, 2521)	2893 (2604, 3182)	3134 (2750, 3519)	3293 (2885, 3701)	2432 (2362, 2502)	3742 (3134, 4349)	5515 (4238, 6792)	7731 (5658, 9803)
**Spain**	33119 (23658, 42580)	36254 (25414, 47094)	34430 (24230, 44629)	39546 (27973, 51119)	28512 (20778, 36245)	47232 (32275, 62189)	61994 (41457, 82530)	71181 (47745, 94617)
**Sweden**	8014 (6145, 9883)	9875 (7237, 12512)	9383 (6725, 12041)	10684 (7777, 13591)	8389 (6379, 10398)	12895 (9125, 16665)	17178 (11597, 22759)	21722 (14675, 28769)
**Switzerland**	13286 (11265, 15308)	13805 (11345, 16264)	14788 (12201, 17375)	15575 (12746, 18405)	13039 (11111, 14968)	16767 (13196, 20337)	23861 (17871, 29850)	30368 (21991, 38745)
**United Kingdom**	66421 (55446, 77396)	65558 (53966, 77150)	75459 (62526, 88393)	77085 (62891, 91279)	58618 (50569, 66667)	86407 (66997, 105817)	123052 (92271, 153832)	143539 (104425, 182654)
**United States**	79970 (47047, 112894)	93475 (57568, 129382)	81006 (46691, 115320)	94907 (53715, 136099)	81883 (48242, 115524)	116470 (71940, 161000)	132004 (78565, 185443)	143070 (83817, 202323)

***Table S6**: Estimated economic cost associated with the wellbeing burden resulting from warming-related increase in moderate-to-severe obstructive sleep apnea (OSA) prevalence for different countries under different climate models from the intergovernmental panel on climate change. Economic cost (in USD millions) was calculated for each scenario (SSP126, SSP245, SSP370 and SSP585) and for each country in 2050 and 2100.*

	2050				2100			
**country**	**SSP126**	**SSP245**	**SSP370**	**SSP585**	**SSP126**	**SSP245**	**SSP370**	**SSP585**
**total**	81402 (40701, 122102)	87489 (43744, 131233)	89939 (44970, 134909)	98921 (49461, 148382)	80554 (40277, 120830)	112077 (56038, 168115)	147375 (73688, 221063)	180170 (90085, 270256)
**Australia**	7100 (3550, 10650)	7179 (3590, 10769)	7339 (3670, 11009)	7410 (3705, 11116)	7205 (3602, 10807)	7664 (3832, 11495)	8572 (4286, 12857)	9613 (4806, 14419)
**Belgium**	1383 (692, 2075)	1320 (660, 1979)	1537 (769, 2306)	1578 (789, 2367)	1313 (657, 1970)	1690 (845, 2534)	2311 (1155, 3466)	2873 (1437, 4310)
**Bulgaria**	227 (113, 340)	325 (162, 487)	309 (154, 463)	370 (185, 555)	236 (118, 354)	412 (206, 619)	563 (281, 844)	754 (377, 1131)
**Canada**	900 (450, 1350)	1001 (500, 1501)	1053 (526, 1579)	1275 (637, 1912)	890 (445, 1336)	1537 (768, 2305)	1964 (982, 2946)	2519 (1259, 3778)
**Czech Republic**	582 (291, 873)	568 (284, 851)	634 (317, 951)	674 (337, 1011)	570 (285, 855)	712 (356, 1069)	1005 (503, 1508)	1446 (723, 2169)
**Denmark**	914 (457, 1370)	933 (467, 1400)	1014 (507, 1520)	1132 (566, 1698)	893 (446, 1339)	1267 (634, 1901)	1770 (885, 2655)	2300 (1150, 3450)
**Estonia**	49 (25, 74)	60 (30, 90)	58 (29, 87)	61 (31, 92)	53 (27, 80)	72 (36, 107)	90 (45, 135)	111 (55, 166)
**Finland**	425 (213, 638)	475 (237, 712)	462 (231, 693)	562 (281, 842)	466 (233, 698)	651 (325, 976)	892 (446, 1338)	1133 (566, 1699)
**France**	6915 (3457, 10372)	6705 (3353, 10058)	7904 (3952, 11857)	7633 (3817, 11450)	6783 (3391, 10174)	8253 (4127, 12380)	11978 (5989, 17967)	14251 (7126, 21377)
**Germany**	9945 (4973, 14918)	9445 (4722, 14167)	11034 (5517, 16551)	11891 (5945, 17836)	9625 (4812, 14437)	13056 (6528, 19584)	17641 (8820, 26461)	23021 (11510, 34531)
**Greece**	494 (247, 741)	592 (296, 888)	646 (323, 969)	722 (361, 1084)	490 (245, 735)	799 (400, 1199)	1166 (583, 1749)	1467 (734, 2201)
**Hungary**	374 (187, 561)	463 (231, 694)	471 (235, 706)	519 (259, 778)	366 (183, 549)	587 (294, 881)	802 (401, 1203)	1071 (535, 1606)
**Ireland**	684 (342, 1026)	635 (317, 952)	741 (371, 1112)	763 (382, 1145)	660 (330, 990)	938 (469, 1407)	1394 (697, 2091)	1698 (849, 2546)

**Italy**	4398<br>(2199, 6597)	5308<br>(2654, 7962)	5284<br>(2642, 7925)	5994<br>(2997, 8992)	4353<br>(2176, 6529)	6648<br>(3324, 9972)	8985<br>(4492, 13477)	11208<br>(5604, 16812)
**Japan**	9671<br>(4835, 14506)	11854<br>(5927, 17781)	11275<br>(5637, 16912)	13470<br>(6735, 20205)	10243<br>(5122, 15365)	15099<br>(7550, 22649)	19946<br>(9973, 29919)	25474<br>(12737, 38210)
**Luxembourg**	233<br>(117, 350)	234<br>(117, 351)	276<br>(138, 414)	270<br>(135, 405)	218<br>(109, 328)	304<br>(152, 456)	432<br>(216, 648)	505<br>(252, 757)
**Mexico**	3363<br>(1682, 5045)	3522<br>(1761, 5282)	4010<br>(2005, 6015)	4192<br>(2096, 6289)	3527<br>(1763, 5290)	4950<br>(2475, 7425)	7243<br>(3622, 10865)	9637<br>(4819, 14456)
**Netherlands**	3155<br>(1577, 4732)	3103<br>(1551, 4654)	3533<br>(1767, 5300)	3778<br>(1889, 5667)	3000<br>(1500, 4499)	4100<br>(2050, 6150)	5519<br>(2760, 8279)	6785<br>(3393, 10178)
**Norway**	636<br>(318, 954)	722<br>(361, 1083)	755<br>(377, 1132)	881<br>(441, 1322)	661<br>(331, 992)	1035<br>(518, 1553)	1432<br>(716, 2148)	1826<br>(913, 2739)
**Poland**	1614<br>(807, 2421)	1667<br>(833, 2500)	1813<br>(906, 2719)	1986<br>(993, 2979)	1550<br>(775, 2325)	2257<br>(1128, 3385)	2818<br>(1409, 4227)	3975<br>(1988, 5963)
**Portugal**	530<br>(265, 795)	558<br>(279, 837)	579<br>(290, 869)	617<br>(309, 926)	465<br>(232, 697)	728<br>(364, 1093)	1089<br>(545, 1634)	1304<br>(652, 1956)
**Romania**	454<br>(227, 681)	595<br>(298, 893)	551<br>(275, 826)	613<br>(306, 919)	445<br>(223, 668)	703<br>(351, 1054)	878<br>(439, 1317)	1118<br>(559, 1678)
**Russia**	891<br>(446, 1337)	948<br>(474, 1422)	534<br>(267, 802)	1163<br>(581, 1744)	835<br>(418, 1253)	1539<br>(769, 2308)	2462<br>(1231, 3694)	3133<br>(1566, 4699)
**Slovakia**	124<br>(62, 186)	147<br>(73, 220)	159<br>(79, 238)	167<br>(83, 250)	123<br>(62, 185)	190<br>(95, 285)	280<br>(140, 419)	392<br>(196, 588)
**Spain**	2205<br>(1103, 3308)	2414<br>(1207, 3621)	2292<br>(1146, 3439)	2633<br>(1317, 3950)	1898<br>(949, 2847)	3145<br>(1572, 4717)	4128<br>(2064, 6191)	4739<br>(2370, 7109)
**Sweden**	915<br>(458, 1373)	1128<br>(564, 1691)	1071<br>(536, 1607)	1220<br>(610, 1830)	958<br>(479, 1437)	1473<br>(736, 2209)	1962<br>(981, 2943)	2481<br>(1240, 3721)
**Switzerland**	2693<br>(1347, 4040)	2798<br>(1399, 4198)	2998<br>(1499, 4497)	3157<br>(1579, 4736)	2643<br>(1322, 3965)	3399<br>(1699, 5098)	4837<br>(2419, 7256)	6156<br>(3078, 9234)
**United Kingdom**	6605<br>(3303, 9908)	6520<br>(3260, 9779)	7504<br>(3752, 11256)	7666<br>(3833, 11499)	5829<br>(2915, 8744)	8593<br>(4297, 12890)	12237<br>(6119, 18356)	14275<br>(7137, 21412)
**United States**	13922<br>(6961, 20883)	16273<br>(8137, 24410)	14102<br>(7051, 21153)	16522<br>(8261, 24783)	14255<br>(7128, 21383)	20276<br>(10138, 30414)	22981<br>(11490, 34471)	24907<br>(12454, 37361)

Table S7: *Projected wellbeing burden of warming-related increase in moderate-to-severe obstructive sleep apnea (OSA) prevalence for different countries under different scenarios from the intergovernmental panel on climate change as well as different modelling assumptions. Disability-adjusted life years (DALY) rates (per 100,000 persons) were calculated for each scenario (SSP245, SSP370) and for each country in 2050 and 2100. Alternative model accounts for population forecast between 2023 and 2100 and assumes a 30% body mass index-related increase in OSA prevalence between 2023 and 2050.*

	DALY per 100,000 persons (2050)				DALY per 100,000 persons (2100)			
**country**	**SSP245** – main	**SSP245 - alternative**	**SSP370** – main	**SSP370 - alternative**	**SSP245-** main	**SSP245 - alternative**	**SSP370** – main	**SSP370 - alternative**
**total**	126 (101, 151)	144 (111, 177)	133 (106, 159)	151 (116, 186)	162 (123, 201)	199 (148, 250)	133 (106, 159)	275 (196, 355)
**Australia**	276 (260, 292)	238 (217, 258)	282 (265, 299)	244 (222, 266)	295 (272, 317)	217 (187, 247)	282 (265, 299)	260 (215, 306)
**Belgium**	135 (112, 158)	149 (119, 179)	157 (131, 184)	174 (139, 209)	173 (136, 210)	203 (154, 252)	157 (131, 184)	283 (209, 358)
**Bulgaria**	188 (140, 236)	243 (180, 306)	179 (129, 228)	232 (166, 298)	238 (171, 306)	348 (259, 436)	179 (129, 228)	455 (315, 594)
**Canada**	30 (18, 42)	40 (24, 55)	32 (19, 45)	43 (26, 60)	46 (28, 64)	61 (38, 85)	32 (19, 45)	79 (48, 110)
**Czech Republic**	109 (99, 119)	123 (110, 136)	122 (110, 134)	138 (122, 155)	137 (117, 157)	176 (149, 203)	122 (110, 134)	250 (198, 302)
**Denmark**	147 (114, 180)	171 (128, 215)	160 (124, 196)	186 (139, 234)	200 (147, 253)	242 (173, 312)	160 (124, 196)	346 (239, 453)
**Estonia**	94 (78, 109)	112 (92, 133)	91 (76, 106)	109 (89, 128)	112 (89, 134)	159 (129, 188)	91 (76, 106)	196 (151, 241)
**Finland**	99 (88, 110)	109 (94, 124)	96 (88, 105)	104 (93, 116)	136 (111, 161)	168 (134, 201)	96 (88, 105)	233 (177, 289)
**France**	147 (128, 166)	157 (133, 182)	174 (151, 197)	187 (156, 217)	181 (150, 213)	200 (158, 242)	174 (151, 197)	302 (227, 377)
**Germany**	130 (104, 156)	155 (121, 190)	152 (121, 182)	182 (142, 222)	180 (135, 224)	229 (170, 288)	152 (121, 182)	311 (225, 396)
**Greece**	155 (106, 203)	199 (135, 263)	169 (117, 220)	217 (149, 285)	209 (140, 278)	283 (192, 374)	169 (117, 220)	411 (275, 547)
**Hungary**	134 (95, 173)	170 (118, 221)	137 (96, 177)	173 (119, 227)	170 (118, 223)	222 (153, 292)	137 (96, 177)	305 (203, 407)
**Ireland**	79 (77, 81)	68 (65, 71)	93 (90, 95)	79 (76, 82)	117 (101, 134)	122 (100, 144)	93 (90, 95)	192 (148, 236)

**Italy**	140<br>(97, 183)	179<br>(122, 236)	139<br>(98, 181)	177<br>(122, 233)	175<br>(119, 232)	238<br>(163, 313)	139<br>(98, 181)	321<br>(217, 424)
**Japan**	168<br>(124, 213)	214<br>(155, 272)	160<br>(115, 205)	204<br>(145, 263)	214<br>(152, 276)	295<br>(214, 377)	160<br>(115, 205)	384<br>(264, 504)
**Luxembourg**	173<br>(150, 197)	172<br>(141, 204)	205<br>(176, 234)	205<br>(167, 243)	226<br>(182, 269)	245<br>(187, 302)	205<br>(176, 234)	362<br>(266, 458)
**Mexico**	147<br>(123, 170)	144<br>(114, 175)	167<br>(140, 193)	165<br>(130, 200)	206<br>(160, 252)	226<br>(165, 287)	167<br>(140, 193)	347<br>(243, 450)
**Netherlands**	173<br>(137, 210)	198<br>(149, 246)	197<br>(156, 238)	224<br>(171, 278)	229<br>(171, 286)	275<br>(199, 351)	197<br>(156, 238)	376<br>(267, 485)
**Norway**	96<br>(75, 117)	108<br>(81, 135)	100<br>(79, 122)	113<br>(85, 141)	138<br>(101, 174)	166<br>(118, 214)	100<br>(79, 122)	235<br>(162, 308)
**Poland**	122<br>(97, 147)	149<br>(116, 182)	132<br>(103, 161)	163<br>(125, 201)	165<br>(123, 206)	246<br>(191, 300)	132<br>(103, 161)	300<br>(225, 375)
**Portugal**	119<br>(89, 150)	147<br>(107, 187)	124<br>(93, 155)	153<br>(111, 194)	156<br>(112, 200)	201<br>(142, 259)	124<br>(93, 155)	302<br>(206, 398)
**Romania**	108<br>(74, 142)	139<br>(94, 184)	100<br>(66, 134)	129<br>(85, 174)	127<br>(86, 169)	174<br>(119, 228)	100<br>(66, 134)	213<br>(138, 287)
**Russia**	31<br>(28, 33)	33<br>(30, 36)	17<br>(20, 15)	16<br>(19, 13)	50<br>(40, 59)	60<br>(48, 73)	17<br>(20, 15)	100<br>(71, 128)
**Slovakia**	69<br>(62, 76)	78<br>(69, 87)	74<br>(65, 84)	86<br>(74, 98)	89<br>(74, 103)	127<br>(108, 146)	74<br>(65, 84)	182<br>(142, 223)
**Spain**	95<br>(66, 123)	119<br>(81, 156)	90<br>(63, 117)	112<br>(77, 148)	123<br>(84, 163)	163<br>(111, 215)	90<br>(63, 117)	214<br>(143, 285)
**Sweden**	123<br>(90, 156)	147<br>(103, 190)	117<br>(84, 150)	141<br>(97, 185)	161<br>(114, 208)	196<br>(133, 258)	117<br>(84, 150)	269<br>(177, 362)
**Switzerland**	197<br>(162, 232)	218<br>(171, 264)	211<br>(174, 248)	233<br>(184, 282)	239<br>(188, 290)	277<br>(209, 344)	211<br>(174, 248)	407<br>(294, 520)
**United Kingdom**	126<br>(104, 149)	136<br>(107, 166)	146<br>(120, 171)	156<br>(123, 190)	167<br>(129, 204)	189<br>(139, 239)	146<br>(120, 171)	277<br>(198, 356)
**United States**	38<br>(23, 53)	50<br>(31, 70)	33<br>(19, 47)	45<br>(26, 64)	48<br>(29, 66)	63<br>(38, 88)	33<br>(19, 47)	73<br>(44, 103)

Table S8: *Projected wellbeing burden of warming-related increase in moderate-to-severe obstructive sleep apnea (OSA) prevalence for different countries under different scenarios from the intergovernmental panel on climate change as well as different modelling assumptions. Disability-adjusted life years DALY were calculated for each scenario (SSP245, SSP370) and for each country in 2050 and 2100. Alternative model accounts for population forecast between 2023 and 2100 and assumes a 30% body mass index-related increase in OSA prevalence between 2023 and 2050.*

	DALY (2050)				DALY (2100)			
**country**	**SSP245** – main	**SSP245 - alternative**	**SSP370** – main	**SSP370 - alternative**	**SSP245-** main	**SSP245 - alternative**	**SSP370** – main	**SSP370 - alternative**
**total**	1029617 (800714, 1258521)	1230918 (923475, 1538361)	1060466 (826503, 1294429)	1270174 (954641, 1585707)	1343064 (995065, 1691064)	1546201 (1118513, 1973888)	1060466 (826503, 1294429)	2122848 (1482558, 2763138)
**Australia**	55472 (52326, 58618)	61065 (55755, 66376)	56708 (53333, 60082)	62729 (57033, 68425)	59214 (54650, 63777)	74109 (63858, 84360)	56708 (53333, 60082)	89092 (73410, 104774)
**Belgium**	12338 (10259, 14416)	14447 (11544, 17350)	14375 (11962, 16788)	16852 (13482, 20223)	15798 (12409, 19186)	18609 (14130, 23088)	14375 (11962, 16788)	25933 (19124, 32743)
**Bulgaria**	10273 (7651, 12896)	10677 (7897, 13458)	9777 (7051, 12503)	10194 (7305, 13084)	13055 (9375, 16735)	10231 (7620, 12842)	9777 (7051, 12503)	13380 (9279, 17481)
**Canada**	9375 (5736, 13014)	14824 (9070, 20578)	9861 (5850, 13871)	15867 (9526, 22208)	14399 (8855, 19942)	26695 (16440, 36949)	9861 (5850, 13871)	34503 (21096, 47910)
**Czech Republic**	9327 (8490, 10164)	9847 (8810, 10883)	10422 (9366, 11478)	11081 (9775, 12388)	11707 (9969, 13446)	11909 (10092, 13727)	10422 (9366, 11478)	16920 (13403, 20438)
**Denmark**	6865 (5328, 8401)	8425 (6281, 10568)	7456 (5776, 9136)	9170 (6826, 11514)	9322 (6859, 11786)	11616 (8268, 14963)	7456 (5776, 9136)	16570 (11434, 21707)
**Estonia**	1008 (842, 1174)	1092 (894, 1290)	974 (812, 1136)	1056 (862, 1249)	1201 (962, 1440)	1111 (906, 1316)	974 (812, 1136)	1373 (1059, 1686)
**Finland**	4415 (3915, 4915)	4820 (4166, 5473)	4296 (3917, 4675)	4606 (4110, 5101)	6055 (4936, 7173)	6489 (5206, 7772)	4296 (3917, 4675)	9035 (6868, 11201)
**France**	75409 (65735, 85083)	85250 (71746, 98755)	88892 (77119, 100665)	101052 (84617, 117487)	92816 (76556, 109076)	111048 (87782, 134314)	88892 (77119, 100665)	167687 (126038, 209335)
**Germany**	89533 (71602, 107464)	100345 (78185, 122504)	104599 (83553, 125645)	117426 (91417, 143436)	123765 (92911, 154619)	132041 (97915, 166167)	104599 (83553, 125645)	179455 (130029, 228882)
**Greece**	12869 (8842, 16895)	14727 (10008, 19447)	14048 (9769, 18327)	16018 (11003, 21033)	17380 (11637, 23124)	15126 (10255, 19997)	14048 (9769, 18327)	21965 (14686, 29244)
**Hungary**	10449 (7409, 13488)	12039 (8380, 15699)	10633 (7446, 13820)	12301 (8465, 16138)	13253 (9151, 17354)	13594 (9346, 17842)	10633 (7446, 13820)	18629 (12397, 24860)
**Ireland**	3061 (2976, 3146)	3204 (3066, 3341)	3575 (3480, 3670)	3747 (3593, 3900)	4522 (3886, 5158)	5374 (4418, 6329)	3575 (3480, 3670)	8446 (6520, 10372)
**Italy**	69165 (47778, 90551)	79183 (53874, 104492)	68846 (48200, 89491)	78521 (54090, 102953)	86622 (58643, 114600)	72368 (49633, 95104)	68846 (48200, 89491)	97437 (65863, 129012)

**Japan**	175176<br>(128740, 221612)	190727<br>(138220, 243234)	166615<br>(120046, 213184)	182143<br>(129486, 234801)	223134<br>(158635, 287633)	193090<br>(139693, 246486)	166615<br>(120046, 213184)	250785<br>(172274, 329295)
**Luxembourg**	911<br>(786, 1037)	1114<br>(910, 1317)	1077<br>(925, 1229)	1326<br>(1079, 1574)	1185<br>(956, 1414)	1518<br>(1161, 1874)	1077<br>(925, 1229)	2244<br>(1649, 2838)
**Mexico**	126436<br>(106411, 146461)	164181<br>(129356, 199006)	143965<br>(121038, 166892)	187532<br>(147661, 227402)	177735<br>(137907, 217563)	244471<br>(178652, 310290)	143965<br>(121038, 166892)	374257<br>(262305, 486210)
**Netherlands**	24809<br>(19566, 30053)	30354<br>(22929, 37778)	28248<br>(22396, 34100)	34502<br>(26216, 42789)	32780<br>(24533, 41027)	39766<br>(28795, 50738)	28248<br>(22396, 34100)	54296<br>(38551, 70042)
**Norway**	4104<br>(3224, 4985)	5198<br>(3894, 6502)	4291<br>(3374, 5207)	5433<br>(4075, 6790)	5884<br>(4333, 7436)	7442<br>(5292, 9592)	4291<br>(3374, 5207)	10535<br>(7254, 13816)
**Poland**	37682<br>(29932, 45432)	41349<br>(32172, 50526)	40990<br>(32019, 49961)	45249<br>(34626, 55872)	51033<br>(38206, 63861)	41504<br>(32268, 50739)	40990<br>(32019, 49961)	50665<br>(37983, 63347)
**Portugal**	10227<br>(7628, 12826)	11780<br>(8576, 14984)	10615<br>(7932, 13298)	12221<br>(8914, 15529)	13352<br>(9571, 17134)	14283<br>(10132, 18434)	10615<br>(7932, 13298)	21526<br>(14699, 28353)
**Romania**	16158<br>(11035, 21281)	17951<br>(12127, 23775)	14948<br>(9850, 20045)	16725<br>(10929, 22520)	19080<br>(12845, 25315)	15647<br>(10700, 20595)	14948<br>(9850, 20045)	19175<br>(12447, 25903)
**Russia**	34314<br>(31772, 36856)	36231<br>(32994, 39468)	19336<br>(22114, 16558)	17461<br>(21000, 13923)	55687<br>(44920, 66453)	61300<br>(48416, 74185)	19336<br>(22114, 16558)	101515<br>(72708, 130322)
**Slovakia**	2997<br>(2694, 3300)	3183<br>(2810, 3557)	3247<br>(2844, 3650)	3494<br>(2998, 3990)	3876<br>(3239, 4512)	3663<br>(3108, 4218)	3247<br>(2844, 3650)	5264<br>(4097, 6432)
**Spain**	36934<br>(25823, 48045)	45134<br>(30841, 59426)	35076<br>(24621, 45531)	42776<br>(29327, 56224)	48119<br>(32787, 63450)	45884<br>(31308, 60459)	35076<br>(24621, 45531)	60174<br>(40161, 80187)
**Sweden**	10014<br>(7327, 12700)	13418<br>(9421, 17415)	9515<br>(6807, 12223)	12937<br>(8908, 16965)	13076<br>(9236, 16917)	18296<br>(12461, 24131)	9515<br>(6807, 12223)	25173<br>(16535, 33811)
**Switzerland**	13993<br>(11489, 16497)	16786<br>(13203, 20370)	14990<br>(12356, 17624)	17942<br>(14173, 21711)	16995<br>(13360, 20631)	20631<br>(15600, 25662)	14990<br>(12356, 17624)	30344<br>(21903, 38784)
**United Kingdom**	66708<br>(54845, 78571)	82328<br>(64408, 100248)	76784<br>(63548, 90020)	94380<br>(74386, 114374)	87924<br>(68059, 107788)	115144<br>(84847, 145441)	76784<br>(63548, 90020)	168753<br>(120708, 216797)
**United States**	99596<br>(60554, 138638)	151238<br>(91936, 210540)	86310<br>(48999, 123621)	135433<br>(78761, 192105)	124097<br>(75679, 172515)	213243<br>(130213, 296272)	86310<br>(48999, 123621)	247717<br>(148076, 347357)

Table S9: *Projected workplace productivity loss and associated economic cost resulting from warming-related increase in moderate-to-severe obstructive sleep apnea (OSA) prevalence for different countries.*

country	Rate of labor loss per 100,000 people (x1000USD)		Count of presenteeism days (x1000)	Count of absenteeism days (x1000)	Count of labor loss (x1000 USD)
	2000	2023			
**total**	3969.6<br>(2696.2, 5243.0)	8406.4<br>(5709.8, 11103.0)	80256<br>(59012, 101500)	25111<br>(12556, 37667)	30288747<br>(20572671, 40004822)
**Australia**	7598.0<br>(5160.7, 10035.3)	22285.3<br>(15136.6, 29434.1)	6082<br>(4472, 7692)	1903<br>(952, 2855)	2479191<br>(1683912, 3274470)
**Belgium**	6290.2<br>(4272.4, 8307.9)	12186.0<br>(8276.9, 16095.0)	986<br>(725, 1248)	309<br>(154, 463)	519724<br>(353006, 686442)
**Bulgaria**	3448.1<br>(2342.0, 4554.2)	5813.0<br>(3948.3, 7677.7)	655<br>(482, 829)	205<br>(102, 307)	169667<br>(115241, 224094)
**Canada**	656.5<br>(445.9, 867.1)	1119.3<br>(760.2, 1478.3)	514<br>(378, 650)	161<br>(80, 241)	197805<br>(134353, 261257)
**Czech Republic**	3150.3<br>(2139.7, 4160.8)	6418.0<br>(4359.2, 8476.8)	862<br>(634, 1090)	270<br>(135, 405)	300213<br>(203910, 396516)
**Denmark**	6706.7<br>(4555.3, 8858.1)	12351.2<br>(8389.2, 16313.3)	601<br>(442, 760)	188<br>(94, 282)	306174<br>(207959, 404389)
**Estonia**	1946.0<br>(1321.8, 2570.3)	4197.7<br>(2851.1, 5544.2)	90<br>(66, 113)	28<br>(14, 42)	26308<br>(17869, 34747)
**Finland**	2653.3<br>(1802.2, 3504.4)	6143.6<br>(4172.8, 8114.3)	318<br>(234, 402)	99<br>(50, 149)	136921<br>(92999, 180842)
**France**	5028.2<br>(3415.2, 6641.1)	11183.9<br>(7596.3, 14771.5)	6284<br>(4620, 7947)	1966<br>(983, 2949)	2876814<br>(1953985, 3799643)
**Germany**	4944.9<br>(3358.7, 6531.1)	9996.2<br>(6789.6, 13202.8)	8132<br>(5979, 10284)	2544<br>(1272, 3816)	3521791<br>(2392065, 4651516)
**Greece**	3176.2<br>(2157.4, 4195.1)	6393.5<br>(4342.6, 8444.4)	793<br>(583, 1003)	248<br>(124, 372)	258762<br>(175756, 341769)
**Hungary**	2214.8<br>(1504.3, 2925.2)	5495.6<br>(3732.7, 7258.5)	835<br>(614, 1057)	261<br>(131, 392)	243450<br>(165356, 321545)
**Ireland**	5403.7<br>(3670.3, 7137.1)	10884.4<br>(7392.9, 14376.0)	293<br>(215, 370)	92<br>(46, 137)	242565<br>(164755, 320376)
**Italy**	3109.4<br>(2111.9, 4106.8)	8569.3<br>(5820.4, 11318.1)	3881<br>(2853, 4908)	1214<br>(607, 1821)	1823590<br>(1238615, 2408564)

**Japan**	2244.5<br>(1524.5, 2964.5)	6934.1<br>(4709.7, 9158.4)	13645<br>(10033, 17256)	4269<br>(2135, 6404)	4188135<br>(2844658, 5531612)
**Luxembourg**	14042.1<br>(9537.6, 18546.5)	27195.1<br>(18471.4, 35918.8)	82<br>(60, 104)	26<br>(13, 38)	78369<br>(53230, 103508)
**Mexico**	2385.5<br>(1620.3, 3150.8)	3477.5<br>(2362.0, 4593.0)	10290<br>(7566, 13013)	3220<br>(1610, 4829)	1822477<br>(1237860, 2407095)
**Netherlands**	7253.8<br>(4926.9, 9580.7)	13536.3<br>(9194.1, 17878.5)	2034<br>(1496, 2573)	637<br>(318, 955)	940263<br>(638644, 1241882)
**Norway**	5279.0<br>(3585.6, 6972.4)	8977.7<br>(6097.8, 11857.6)	329<br>(242, 416)	103<br>(51, 154)	203370<br>(138133, 268607)
**Poland**	3403.8<br>(2311.9, 4495.6)	6658.0<br>(4522.2, 8793.8)	3375<br>(2482, 4268)	1056<br>(528, 1584)	1097711<br>(745585, 1449836)
**Portugal**	2337.9<br>(1587.9, 3087.8)	4875.8<br>(3311.8, 6439.9)	717<br>(527, 906)	224<br>(112, 336)	223299<br>(151669, 294930)
**Romania**	1295.2<br>(879.7, 1710.7)	4957.3<br>(3367.1, 6547.5)	1097<br>(807, 1388)	343<br>(172, 515)	389889<br>(264820, 514959)
**Russia**	-275.0<br>(-186.8, -363.2)	535.0<br>(363.4, 706.6)	1250<br>(919, 1580)	391<br>(196, 587)	372825<br>(253230, 492421)
**Slovakia**	1683.6<br>(1143.6, 2223.7)	2643.4<br>(1795.4, 3491.3)	222<br>(163, 281)	70<br>(35, 104)	64058<br>(43509, 84606)
**Spain**	2670.3<br>(1813.7, 3526.8)	4724.5<br>(3209.0, 6240.1)	2462<br>(1811, 3114)	770<br>(385, 1156)	937669<br>(636882, 1238456)
**Sweden**	4000.6<br>(2717.3, 5283.9)	7963.1<br>(5408.7, 10517.5)	785<br>(577, 993)	246<br>(123, 368)	355301<br>(241327, 469275)
**Switzerland**	7181.8<br>(4878.0, 9485.5)	17574.6<br>(11937.0, 23212.2)	1327<br>(976, 1679)	415<br>(208, 623)	729872<br>(495743, 964002)
**United Kingdom**	4466.2<br>(3033.5, 5898.9)	8377.8<br>(5690.3, 11065.2)	5953<br>(4377, 7528)	1863<br>(931, 2794)	2347822<br>(1594684, 3100961)
**United States**	822.7<br>(558.8, 1086.6)	2318.6<br>(1574.8, 3062.3)	6363<br>(4678, 8047)	1991<br>(995, 2986)	3434710<br>(2332918, 4536502)

Table S10: *Estimated economic cost associated with the labor loss resulting from warming-related increase in moderate-to-severe obstructive sleep apnea (OSA) prevalence for different countries under different climate models from the intergovernmental panel on climate change. Economic cost (in USD millions) was calculated for each scenario (SSP126, SSP245, SSP370 and SSP585) and for each country in 2050 and 2100.*

	2050				2100			
**country**	**SSP126**	**SSP245**	**SSP370**	**SSP585**	**SSP126**	**SSP245**	**SSP370**	**SSP585**
**total**	35702 (24249, 47155)	39455 (26799, 52111)	41144 (27946, 54342)	45034 (30588, 59480)	37206 (25271, 49140)	50544 (34330, 66757)	68567 (46572, 90562)	84271 (57238, 111304)
**Australia**	2573 (1748, 3398)	2588 (1758, 3419)	2653 (1802, 3504)	2678 (1819, 3537)	2597 (1764, 3430)	2765 (1878, 3652)	3072 (2087, 4058)	3363 (2284, 4442)
**Belgium**	580 (394, 766)	580 (394, 765)	652 (443, 861)	682 (463, 901)	578 (392, 763)	706 (479, 932)	992 (674, 1310)	1207 (820, 1594)
**Bulgaria**	215 (146, 284)	262 (178, 346)	253 (172, 334)	307 (208, 405)	224 (152, 296)	350 (238, 463)	472 (321, 624)	620 (421, 819)
**Canada**	305 (207, 402)	369 (251, 487)	370 (251, 488)	480 (326, 634)	331 (225, 437)	545 (370, 720)	767 (521, 1013)	980 (666, 1295)
**Czech Republic**	351 (238, 463)	353 (240, 466)	371 (252, 490)	415 (282, 548)	358 (243, 473)	433 (294, 572)	622 (423, 822)	856 (581, 1130)
**Denmark**	354 (240, 467)	388 (263, 512)	407 (277, 538)	466 (316, 615)	373 (253, 492)	501 (340, 662)	699 (475, 924)	911 (619, 1203)
**Estonia**	29 (20, 38)	33 (22, 43)	33 (22, 43)	35 (24, 47)	30 (21, 40)	40 (27, 53)	50 (34, 67)	63 (43, 83)
**Finland**	171 (116, 226)	191 (130, 252)	202 (137, 267)	227 (154, 300)	186 (126, 246)	255 (174, 337)	354 (240, 467)	450 (305, 594)
**France**	3239 (2200, 4278)	3239 (2200, 4278)	3689 (2506, 4872)	3730 (2534, 4927)	3267 (2219, 4316)	3940 (2676, 5204)	5632 (3825, 7438)	6731 (4572, 8891)
**Germany**	4049 (2750, 5348)	4161 (2826, 5496)	4556 (3094, 6017)	4980 (3383, 6578)	4189 (2845, 5532)	5262 (3574, 6950)	7341 (4986, 9696)	9415 (6395, 12436)
**Greece**	333 (226, 439)	375 (255, 495)	412 (280, 545)	460 (312, 607)	355 (241, 469)	520 (353, 687)	767 (521, 1013)	948 (644, 1252)
**Hungary**	296 (201, 391)	337 (229, 445)	338 (230, 447)	381 (259, 503)	315 (214, 416)	427 (290, 564)	591 (401, 781)	773 (525, 1021)
**Ireland**	275 (187, 363)	289 (196, 382)	340 (231, 450)	345 (234, 456)	272 (185, 360)	404 (274, 533)	602 (409, 795)	724 (492, 957)
**Italy**	2192 (1489, 2895)	2476 (1682, 3270)	2642 (1794, 3489)	2944 (1999, 3888)	2331 (1583, 3078)	3212 (2182, 4243)	4534 (3080, 5989)	5646 (3835, 7458)

**Japan**	4728<br>(3211, 6244)	5828<br>(3959, 7698)	5668<br>(3850, 7486)	6771<br>(4599, 8943)	5032<br>(3418, 6646)	7681<br>(5217, 10144)	10305<br>(6999, 13610)	13023<br>(8846, 17201)
**Luxembourg**	88<br>(60, 117)	92<br>(62, 121)	105<br>(72, 139)	107<br>(73, 141)	90<br>(61, 119)	115<br>(78, 151)	165<br>(112, 218)	194<br>(131, 256)
**Mexico**	2248<br>(1527, 2969)	2476<br>(1682, 3271)	2780<br>(1888, 3671)	2817<br>(1913, 3720)	2333<br>(1585, 3081)	3379<br>(2295, 4463)	5125<br>(3481, 6769)	6313<br>(4288, 8339)
**Netherlands**	1035<br>(703, 1367)	1080<br>(734, 1426)	1200<br>(815, 1585)	1280<br>(870, 1691)	1039<br>(705, 1372)	1330<br>(904, 1757)	1860<br>(1263, 2456)	2277<br>(1546, 3007)
**Norway**	240<br>(163, 316)	279<br>(189, 368)	296<br>(201, 391)	341<br>(232, 451)	258<br>(176, 341)	380<br>(258, 502)	526<br>(358, 695)	665<br>(452, 879)
**Poland**	1257<br>(854, 1661)	1342<br>(912, 1773)	1391<br>(945, 1837)	1577<br>(1071, 2083)	1299<br>(883, 1716)	1699<br>(1154, 2244)	2225<br>(1511, 2939)	3010<br>(2045, 3976)
**Portugal**	273<br>(186, 361)	309<br>(210, 409)	326<br>(222, 431)	354<br>(241, 468)	268<br>(182, 355)	399<br>(271, 528)	596<br>(405, 787)	728<br>(494, 961)
**Romania**	460<br>(312, 608)	543<br>(369, 717)	516<br>(351, 682)	603<br>(409, 796)	472<br>(321, 624)	680<br>(462, 898)	853<br>(579, 1126)	1073<br>(729, 1418)
**Russia**	865<br>(588, 1143)	1139<br>(774, 1505)	1179<br>(801, 1557)	1450<br>(985, 1915)	1069<br>(726, 1412)	1937<br>(1315, 2558)	2973<br>(2019, 3926)	3994<br>(2713, 5275)
**Slovakia**	82<br>(56, 108)	91<br>(62, 121)	92<br>(62, 121)	105<br>(71, 139)	87<br>(59, 115)	119<br>(81, 157)	177<br>(120, 234)	242<br>(164, 319)
**Spain**	1199<br>(814, 1583)	1302<br>(884, 1719)	1349<br>(917, 1782)	1550<br>(1052, 2047)	1207<br>(820, 1595)	1671<br>(1135, 2207)	2322<br>(1577, 3067)	2678<br>(1819, 3537)
**Sweden**	431<br>(293, 569)	501<br>(340, 662)	492<br>(334, 650)	561<br>(381, 741)	455<br>(309, 601)	654<br>(444, 863)	865<br>(588, 1143)	1093<br>(742, 1444)
**Switzerland**	833<br>(566, 1100)	863<br>(586, 1140)	922<br>(626, 1218)	999<br>(679, 1320)	849<br>(577, 1122)	1057<br>(718, 1395)	1490<br>(1012, 1969)	1909<br>(1296, 2521)
**United Kingdom**	2551<br>(1733, 3369)	2755<br>(1871, 3639)	3070<br>(2085, 4055)	3193<br>(2169, 4217)	2381<br>(1617, 3145)	3449<br>(2343, 4556)	4874<br>(3310, 6437)	5712<br>(3879, 7544)
**United States**	4451<br>(3023, 5879)	5214<br>(3541, 6886)	4840<br>(3288, 6393)	5196<br>(3529, 6863)	4959<br>(3368, 6550)	6633<br>(4505, 8761)	7714<br>(5239, 10188)	8674<br>(5891, 11456)

Table S11: *Estimated economic cost associated with the labor loss resulting from warming-related increase in moderate-to-severe obstructive sleep apnea (OSA) prevalence for different countries under different climate models from the intergovernmental panel on climate change. Economic cost (in USD millions) per 100,000 persons was calculated for each scenario (SSP126, SSP245, SSP370 and SSP585) and for each country in 2050 and 2100.*

	2050				2100			
**country**	**SSP126**	**SSP245**	**SSP370**	**SSP585**	**SSP126**	**SSP245**	**SSP370**	**SSP585**
**total**	9.7<br>(6.6, 12.8)	10.5<br>(7.1, 13.8)	11.2<br>(7.6, 14.8)	12.2<br>(8.3, 16.1)	10.0<br>(6.8, 13.2)	13.3<br>(9.0, 17.5)	18.4<br>(12.5, 24.3)	22.8<br>(15.5, 30.1)
**Australia**	23.1<br>(15.7, 30.5)	23.3<br>(15.8, 30.7)	23.8<br>(16.2, 31.5)	24.1<br>(16.4, 31.8)	23.3<br>(15.9, 30.8)	24.9<br>(16.9, 32.8)	27.6<br>(18.8, 36.5)	30.2<br>(20.5, 39.9)
**Belgium**	13.6<br>(9.2, 17.9)	13.6<br>(9.2, 17.9)	15.3<br>(10.4, 20.2)	16.0<br>(10.9, 21.1)	13.5<br>(9.2, 17.9)	16.5<br>(11.2, 21.8)	23.3<br>(15.8, 30.7)	28.3<br>(19.2, 37.4)
**Bulgaria**	7.4<br>(5.0, 9.7)	9.0<br>(6.1, 11.9)	8.7<br>(5.9, 11.4)	10.5<br>(7.1, 13.9)	7.7<br>(5.2, 10.1)	12.0<br>(8.2, 15.9)	16.2<br>(11.0, 21.4)	21.2<br>(14.4, 28.1)
**Canada**	1.7<br>(1.2, 2.3)	2.1<br>(1.4, 2.8)	2.1<br>(1.4, 2.8)	2.7<br>(1.8, 3.6)	1.9<br>(1.3, 2.5)	3.1<br>(2.1, 4.1)	4.3<br>(2.9, 5.7)	5.5<br>(3.8, 7.3)
**Czech Republic**	7.5<br>(5.1, 9.9)	7.5<br>(5.1, 10.0)	7.9<br>(5.4, 10.5)	8.9<br>(6.0, 11.7)	7.7<br>(5.2, 10.1)	9.3<br>(6.3, 12.2)	13.3<br>(9.0, 17.6)	18.3<br>(12.4, 24.2)
**Denmark**	14.3<br>(9.7, 18.8)	15.6<br>(10.6, 20.7)	16.4<br>(11.2, 21.7)	18.8<br>(12.8, 24.8)	15.0<br>(10.2, 19.9)	20.2<br>(13.7, 26.7)	28.2<br>(19.2, 37.3)	36.7<br>(25.0, 48.5)
**Estonia**	4.6<br>(3.1, 6.1)	5.2<br>(3.5, 6.9)	5.2<br>(3.6, 6.9)	5.6<br>(3.8, 7.4)	4.8<br>(3.3, 6.4)	6.4<br>(4.3, 8.4)	8.1<br>(5.5, 10.6)	10.0<br>(6.8, 13.2)
**Finland**	7.7<br>(5.2, 10.2)	8.6<br>(5.8, 11.3)	9.1<br>(6.2, 12.0)	10.2<br>(6.9, 13.5)	8.4<br>(5.7, 11.0)	11.5<br>(7.8, 15.1)	15.9<br>(10.8, 21.0)	20.2<br>(13.7, 26.7)
**France**	12.6<br>(8.6, 16.6)	12.6<br>(8.6, 16.6)	14.3<br>(9.7, 18.9)	14.5<br>(9.8, 19.2)	12.7<br>(8.6, 16.8)	15.3<br>(10.4, 20.2)	21.9<br>(14.9, 28.9)	26.2<br>(17.8, 34.6)
**Germany**	11.5<br>(7.8, 15.2)	11.8<br>(8.0, 15.6)	12.9<br>(8.8, 17.1)	14.1<br>(9.6, 18.7)	11.9<br>(8.1, 15.7)	14.9<br>(10.1, 19.7)	20.8<br>(14.2, 27.5)	26.7<br>(18.2, 35.3)
**Greece**	8.2<br>(5.6, 10.9)	9.3<br>(6.3, 12.2)	10.2<br>(6.9, 13.5)	11.4<br>(7.7, 15.0)	8.8<br>(6.0, 11.6)	12.9<br>(8.7, 17.0)	18.9<br>(12.9, 25.0)	23.4<br>(15.9, 30.9)
**Hungary**	6.7<br>(4.5, 8.8)	7.6<br>(5.2, 10.1)	7.6<br>(5.2, 10.1)	8.6<br>(5.8, 11.4)	7.1<br>(4.8, 9.4)	9.6<br>(6.5, 12.7)	13.3<br>(9.1, 17.6)	17.5<br>(11.9, 23.1)
**Ireland**	12.3<br>(8.4, 16.3)	13.0<br>(8.8, 17.1)	15.3<br>(10.4, 20.2)	15.5<br>(10.5, 20.5)	12.2<br>(8.3, 16.1)	18.1<br>(12.3, 23.9)	27.0<br>(18.3, 35.7)	32.5<br>(22.1, 42.9)
**Italy**	10.3<br>(7.0, 13.6)	11.6<br>(7.9, 15.4)	12.4<br>(8.4, 16.4)	13.8<br>(9.4, 18.3)	11.0<br>(7.4, 14.5)	15.1<br>(10.3, 19.9)	21.3<br>(14.5, 28.1)	26.5<br>(18.0, 35.0)

**Japan**	7.8 (5.3, 10.3)	9.6 (6.6, 12.7)	9.4 (6.4, 12.4)	11.2 (7.6, 14.8)	8.3 (5.7, 11.0)	12.7 (8.6, 16.8)	17.1 (11.6, 22.5)	21.6 (14.6, 28.5)
**Luxembourg**	30.6 (20.8, 40.4)	31.8 (21.6, 42.0)	36.5 (24.8, 48.3)	37.1 (25.2, 48.9)	31.2 (21.2, 41.2)	39.8 (27.0, 52.6)	57.4 (39.0, 75.8)	67.2 (45.6, 88.7)
**Mexico**	4.3 (2.9, 5.7)	4.7 (3.2, 6.2)	5.3 (3.6, 7.0)	5.4 (3.7, 7.1)	4.5 (3.0, 5.9)	6.4 (4.4, 8.5)	9.8 (6.6, 12.9)	12.0 (8.2, 15.9)
**Netherlands**	14.9 (10.1, 19.7)	15.5 (10.6, 20.5)	17.3 (11.7, 22.8)	18.4 (12.5, 24.3)	15.0 (10.2, 19.7)	19.2 (13.0, 25.3)	26.8 (18.2, 35.4)	32.8 (22.3, 43.3)
**Norway**	10.6 (7.2, 14.0)	12.3 (8.4, 16.2)	13.1 (8.9, 17.3)	15.1 (10.2, 19.9)	11.4 (7.7, 15.1)	16.8 (11.4, 22.2)	23.2 (15.8, 30.7)	29.4 (19.9, 38.8)
**Poland**	7.6 (5.2, 10.1)	8.1 (5.5, 10.8)	8.4 (5.7, 11.1)	9.6 (6.5, 12.6)	7.9 (5.4, 10.4)	10.3 (7.0, 13.6)	13.5 (9.2, 17.8)	18.3 (12.4, 24.1)
**Portugal**	6.0 (4.1, 7.9)	6.8 (4.6, 8.9)	7.1 (4.8, 9.4)	7.7 (5.3, 10.2)	5.9 (4.0, 7.7)	8.7 (5.9, 11.5)	13.0 (8.8, 17.2)	15.9 (10.8, 21.0)
**Romania**	5.8 (4.0, 7.7)	6.9 (4.7, 9.1)	6.6 (4.5, 8.7)	7.7 (5.2, 10.1)	6.0 (4.1, 7.9)	8.6 (5.9, 11.4)	10.8 (7.4, 14.3)	13.6 (9.3, 18.0)
**Russia**	1.2 (0.8, 1.6)	1.6 (1.1, 2.2)	1.7 (1.1, 2.2)	2.1 (1.4, 2.7)	1.5 (1.0, 2.0)	2.8 (1.9, 3.7)	4.3 (2.9, 5.6)	5.7 (3.9, 7.6)
**Slovakia**	3.4 (2.3, 4.5)	3.8 (2.6, 5.0)	3.8 (2.6, 5.0)	4.3 (2.9, 5.7)	3.6 (2.4, 4.8)	4.9 (3.3, 6.5)	7.3 (5.0, 9.7)	10.0 (6.8, 13.2)
**Spain**	6.0 (4.1, 8.0)	6.6 (4.5, 8.7)	6.8 (4.6, 9.0)	7.8 (5.3, 10.3)	6.1 (4.1, 8.0)	8.4 (5.7, 11.1)	11.7 (7.9, 15.5)	13.5 (9.2, 17.8)
**Sweden**	9.7 (6.6, 12.8)	11.2 (7.6, 14.8)	11.0 (7.5, 14.6)	12.6 (8.5, 16.6)	10.2 (6.9, 13.5)	14.7 (10.0, 19.4)	19.4 (13.2, 25.6)	24.5 (16.6, 32.4)
**Switzerland**	20.1 (13.6, 26.5)	20.8 (14.1, 27.5)	22.2 (15.1, 29.3)	24.1 (16.3, 31.8)	20.4 (13.9, 27.0)	25.4 (17.3, 33.6)	35.9 (24.4, 47.4)	46.0 (31.2, 60.7)
**United Kingdom**	9.1 (6.2, 12.0)	9.8 (6.7, 13.0)	11.0 (7.4, 14.5)	11.4 (7.7, 15.0)	8.5 (5.8, 11.2)	12.3 (8.4, 16.3)	17.4 (11.8, 23.0)	20.4 (13.8, 26.9)
**United States**	3.0 (2.0, 4.0)	3.5 (2.4, 4.6)	3.3 (2.2, 4.3)	3.5 (2.4, 4.6)	3.3 (2.3, 4.4)	4.5 (3.0, 5.9)	5.2 (3.5, 6.9)	5.9 (4.0, 7.7)

Supplementary methods

Assessment of weather variables and climate projections

We extracted hourly air temperature at 2m from the ground for each of the main cities (square of 500x500km around the location) in the user database for the period 1950–2023 from the fifth generation of European Reanalysis (ERA5) dataset[3] using Copernicus Climate Change Service[4]. ERA5 is a climate reanalysis product and offers land-surface data that have previously been shown to provide a satisfactory proxy to station-based series and used extensively to assess the effect of ambient temperature on health[5,6]. Minimum, maximum, and mean 24h temperatures were calculated for each location. We also extracted hourly dew point temperature, total cloud cover, and surface pressure for each location, which were subsequently averaged over a 24h period. Python (version 3.11), Pandas (version 2.2.1), Xarray (v2024.2.0) and MetPy[7] (version 1.6.2) were used for data manipulation and weather extraction/calculations. Relative humidity and heat index were calculated based on an existing formula[8].

We extracted fine particulate matter (aerodynamic diameter <2.5 µm) concentration from the ECMWF Atmospheric Composition Reanalysis 4 model as a measure of air quality[9]. This model is the fourth-generation global reanalysis of atmospheric composition and combines model data with observations worldwide to create a consistent dataset spanning over a decade that has been validated against station-based measurements[10], and has been used in previous research on the effect of air pollution on health[11].

We used the climate scenarios CMIP6[12] from the 2021 sixth assessment report of the IPCC[13] – called Shared Socioeconomic Pathway (SSP). We downloaded daily temperatures from 2023 to 2100 projections for 4 SSPs (SSP126, SSP245, SSP370, SSP585), based on 27 climate models from multiple countries and climate modelling groups. 4 models were excluded due to missing historical baseline data. For the remaining 23 models, we extracted the daily average temperature at 2m from the ground for each of the main cities (square of 500x500km around the location). Each model was bias corrected to the ERA5 dataset and rescaled using linear scaling correction. Averaged daily projections across models were then calculated as the daily median of the 23 projections and the ensemble median was used for all wellbeing and economics modelling.

Statistical analyses

The exposure-response association of interest between temperature and obstructive sleep apnea (OSA) prevalence was modelled using distributed lags[14,15]. We primarily chose a 4-day lag structure since the effect of temperature on sleep duration was shown to be significant up to the fourth day[16], and we found similar lagged effect in this study (Figure S7). Alternative lag structures were explored but made minimal difference to the main cumulative exposure-response curve (Figure S8).

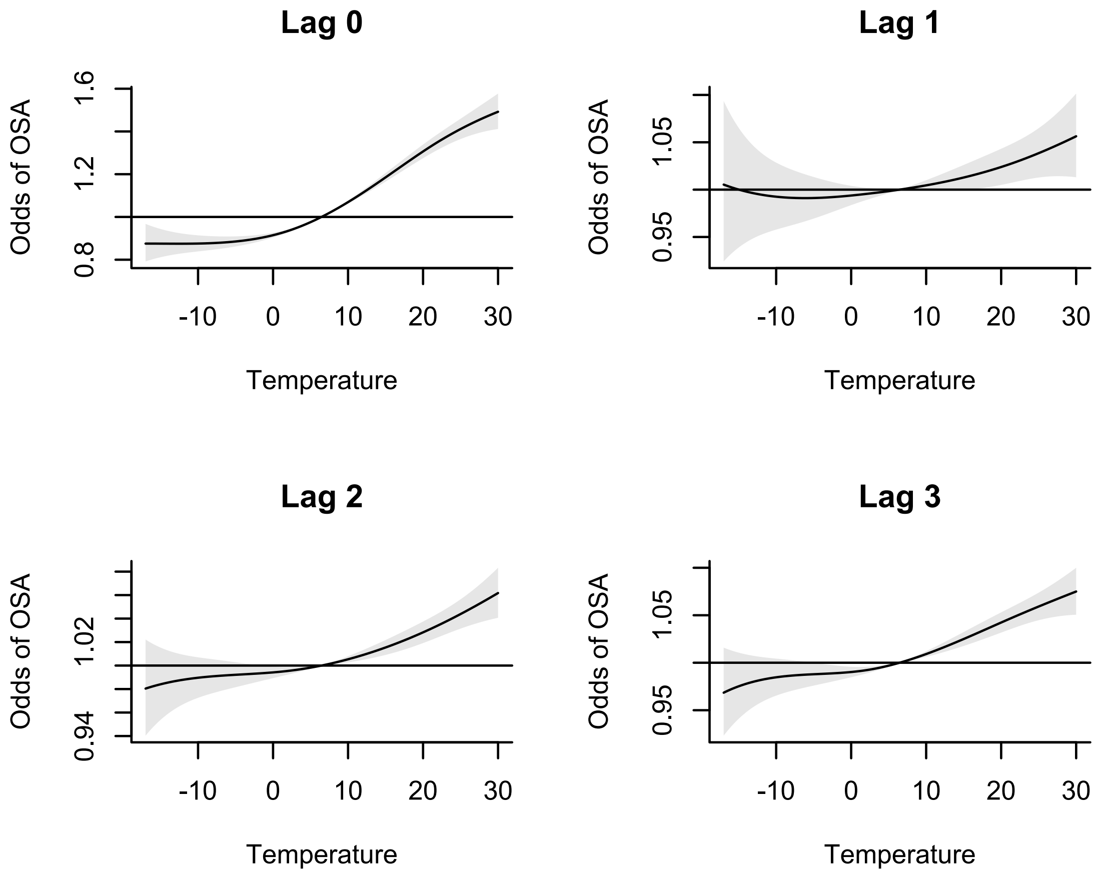

Figure S7: Lagged effect of exposure to temperature on the risk of nightly obstructive sleep apnea (OSA).

Lag 0 represents the acute effect of exposure, lag 1, 2 and 3 represents the effects on the following 1,2 and 3rd day. This analysis was done with a sub-sample of the dataset (N=10,000). Shaded area represents 95%CI.

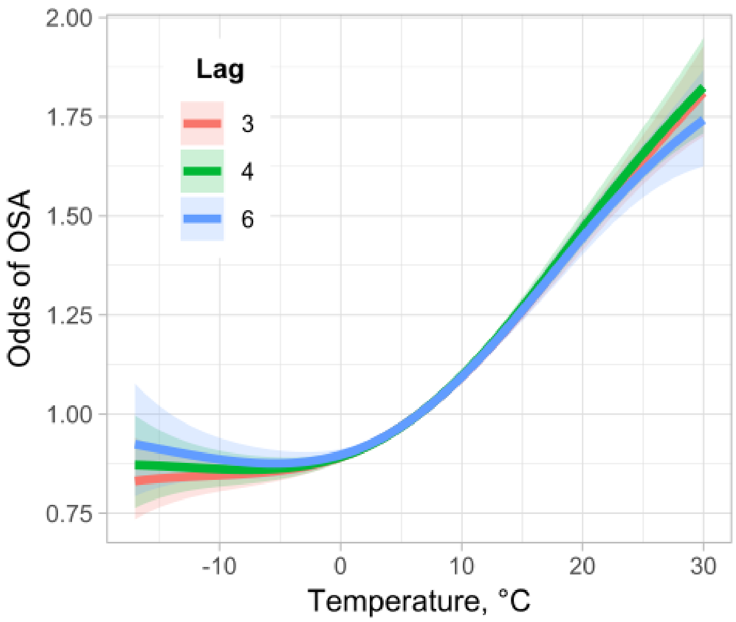

Figure S8: Cumulative lagged exposure-response curve between temperature and risk of nightly obstructive sleep apnea (OSA).

Models were constructed with different number of lags (3, 4 and 6). This analysis was done with a sub-sample of the dataset (N=10,000). Shaded area represents 95%CI.

Supplementary acknowledgments

Table S12: *CMIP6 global climate projections, model names and institutions used in this study and downloaded from the Climate Data Store (CDS) of the Copernicus Climate Change Service (C3S).*

Model name	Institutions
UKESM1-0-LL	Met Office Hadley Centre, UK; Natural Environment Research Council, UK; National Institute of Meteorological Sciences, Korea; National Institute of Water and Atmospheric Research, New Zealand.
EC-Earth3-Veg-LR	AEMET, Spain; BSC, Spain; CNR-ISAC, Italy; DMI, Denmark; ENEA, Italy; FMI, Finland; Geomar, Germany; ICHEC, Ireland; ICTP, Italy; IDL, Portugal; IMAU, The Netherlands; IPMA, Portugal; KIT, Karlsruhe, Germany; KNMI, The Netherlands; Lund University, Sweden; Met Eireann, Ireland; NLeSC, The Netherlands; NTNU, Norway; Oxford University, UK; surfSARA, The Netherlands; SMHI, Sweden; Stockholm University, Sweden; Unite ASTR, Belgium; University College Dublin, Ireland; University of Bergen, Norway; University of Copenhagen, Denmark; University of Helsinki, Finland; University of Santiago de Compostela, Spain; Uppsala University, Sweden; Utrecht University, The Netherlands; Vrije Universiteit Amsterdam, the Netherlands; Wageningen University, The Netherlands. Mailing address: EC-Earth consortium, Rossby Center, Swedish Meteorological and Hydrological Institute/SMHI, SE-601 76 Norrkoping, Sweden
IPSL-CM6A-LR	Institut Pierre Simon Laplace, France
NorESM2-LM	NorESM Climate modeling Consortium consisting of CICERO (Center for International Climate and Environmental Research, Oslo 0349), MET-Norway (Norwegian Meteorological Institute, Oslo 0313), NERSC (Nansen Environmental and Remote Sensing Center, Bergen 5006), NILU (Norwegian Institute for Air Research, Kjeller 2027), UiB (University of Bergen, Bergen 5007), UiO (University of Oslo, Oslo 0313) and UNI (Uni Research, Bergen 5008), Norway.
INM-CM4-8	Institute for Numerical Mathematics, Russian Academy of Science, Russia
MPI-ESM1-2-LR	Max Planck Institute for Meteorology, Germany; Alfred Wegener Institute, Helmholtz Centre for Polar and Marine Research, Germany; Deutsches Klimarechenzentrum, Germany; Deutscher Wetterdienst, Germany
CNRM-CM6-1-HR	CNRM (Centre National de Recherches Meteorologiques, Toulouse 31057, France), CERFACS (Centre Europeen de Recherche et de Formation Avancee en Calcul Scientifique, Toulouse 31057, France)
IITM-ESM	Centre for Climate Change Research, Indian Institute of Tropical Meteorology Pune, Maharashtra 411 008, India
AWI-CM-1-1-MR	Alfred Wegener Institute, Helmholtz Centre for Polar and Marine Research, Am Handelshafen 12, 27570 Bremerhaven, Germany
BCC-CSM2-MR	Beijing Climate Center, Beijing 100081, China
ACCESS-CM2	CSIRO (Commonwealth Scientific and Industrial Research Organisation, Aspendale, Victoria 3195, Australia), ARCCSS (Australian Research Council Centre of Excellence for Climate System Science). Mailing address: CSIRO, c/o Simon J. Marsland, 107-121 Station Street, Aspendale, Victoria 3195, Australia
CNRM-CM6-1	CNRM (Centre National de Recherches Meteorologiques, Toulouse 31057, France), CERFACS (Centre Europeen de Recherche et de Formation Avancee en Calcul Scientifique, Toulouse 31057, France)

CMCC-CM2-SR5	Fondazione Centro Euro-Mediterraneo sui Cambiamenti Climatici, Lecce 73100, Italy
INM-CM5-0	Institute for Numerical Mathematics, Russian Academy of Science, Russia
NorESM2-MM	NorESM Climate modeling Consortium consisting of CICERO (Center for International Climate and Environmental Research, Oslo 0349), MET-Norway (Norwegian Meteorological Institute, Oslo 0313), NERSC (Nansen Environmental and Remote Sensing Center, Bergen 5006), NILU (Norwegian Institute for Air Research, Kjeller 2027), UiB (University of Bergen, Bergen 5007), UiO (University of Oslo, Oslo 0313) and UNI (Uni Research, Bergen 5008), Norway.
GFDL-ESM4	National Oceanic and Atmospheric Administration, Geophysical Fluid Dynamics Laboratory, Princeton, NJ 08540, USA
KACE-1-0-G	National Institute of Meteorological Sciences/Korea Meteorological Administration, Climate Research Division, Seoho-bukro 33, Seogwipo-si, Jejudo 63568, Republic of Korea
CAMS-CSM1-0	Chinese Academy of Meteorological Sciences, Beijing 100081, China
CNRM-ESM2-1	CNRM (Centre National de Recherches Meteorologiques, Toulouse 31057, France), CERFACS (Centre Europeen de Recherche et de Formation Avancee en Calcul Scientifique, Toulouse 31057, France)
MRI-ESM2-0	Meteorological Research Institute, Tsukuba, Ibaraki 305-0052, Japan
HadGEM3-GC31-LL	Met Office Hadley Centre, Fitzroy Road, Exeter, Devon, EX1 3PB, UK; Natural Environment Research Council, STFC-RAL, Harwell, Oxford, OX11 0QX, UK
MIROC6	JAMSTEC (Japan Agency for Marine-Earth Science and Technology, Kanagawa 236-0001, Japan), AORI (Atmosphere and Ocean Research Institute, The University of Tokyo, Chiba 277-8564, Japan), NIES (National Institute for Environmental Studies, Ibaraki 305-8506, Japan), and R-CCS (RIKEN Center for Computational Science, Hyogo 650-0047, Japan)
FGOALS-g3	Chinese Academy of Sciences, Beijing 100029, China
CESM2	National Center for Atmospheric Research, Climate and Global Dynamics Laboratory, 1850 Table Mesa Drive, Boulder, CO 80305, USA
EC-Earth3-AerChem	AEMET, Spain; BSC, Spain; CNR-ISAC, Italy; DMI, Denmark; ENEA, Italy; FMI, Finland; Geomar, Germany; ICHEC, Ireland; ICTP, Italy; IDL, Portugal; IMAU, The Netherlands; IPMA, Portugal; KIT, Karlsruhe, Germany; KNMI, The Netherlands; Lund University, Sweden; Met Eireann, Ireland; NLeSC, The Netherlands; NTNU, Norway; Oxford University, UK; surfSARA, The Netherlands; SMHI, Sweden; Stockholm University, Sweden; Unite ASTR, Belgium; University College Dublin, Ireland; University of Bergen, Norway; University of Copenhagen, Denmark; University of Helsinki, Finland; University of Santiago de Compostela, Spain; Uppsala University, Sweden; Utrecht University, The Netherlands; Vrije Universiteit Amsterdam, the Netherlands; Wageningen University, The Netherlands. Mailing address: EC-Earth consortium, Rossby Center, Swedish Meteorological and Hydrological Institute/SMHI, SE-601 76 Norrkoping, Sweden
CESM2-WACCM	National Center for Atmospheric Research, Climate and Global Dynamics Laboratory, 1850 Table Mesa Drive, Boulder, CO 80305, USA
MPI-ESM-1-2-HAM	ETH Zurich, Switzerland; Max Planck Institut fur Meteorologie, Germany; Forschungszentrum Julich, Germany; University of Oxford, UK; Finnish Meteorological Institute, Finland; Leibniz Institute for Tropospheric Research, Germany; Center for Climate Systems Modeling (C2SM) at ETH Zurich, Switzerland

Supplementary references

1. Edouard P, Campo D, Bartet P, et al. Validation of the Withings Sleep Analyzer, an under-the-mattress device for the detection of moderate-severe sleep apnea syndrome. *J Clin Sleep Med* 2021.
2. Economics DA. The economic cost of sleep disorders in Australia, 2010: Sleep Health Foundation, 2011.
3. Muñoz-Sabater J, Dutra E, Agustí-Panareda A, et al. ERA5-Land: a state-of-the-art global reanalysis dataset for land applications. *Earth System Science Data* 2021; **13**(9): 4349-83.
4. Copernicus Climate Change Service (C3S) Climate Data Store (CDS). Copernicus Climate Change Service (2022): ERA5-Land hourly data from 1950 to present. .
5. Urban A, Di Napoli C, Cloke HL, et al. Evaluation of the ERA5 reanalysis-based Universal Thermal Climate Index on mortality data in Europe. *Environ Res* 2021; **198**: 111227.
6. Mistry MN, Schneider R, Masselot P, et al. Comparison of weather station and climate reanalysis data for modelling temperature-related mortality. *Sci Rep* 2022; **12**(1): 5178.
7. May RM, Goebbert KH, Thielen JE, et al. MetPy: A Meteorological Python Library for Data Analysis and Visualization. *Bulletin of the American Meteorological Society* 2022; **103**(10): E2273-E84.
8. Anderson GB, Bell ML, Peng RD. Methods to calculate the heat index as an exposure metric in environmental health research. *Environ Health Perspect* 2013; **121**(10): 1111-9.
9. Inness A, Ades M, Agustí-Panareda A, et al. The CAMS reanalysis of atmospheric composition. *Atmospheric Chemistry and Physics* 2019; **19**(6): 3515-56.
10. Gueymard CA, Yang D. Worldwide validation of CAMS and MERRA-2 reanalysis aerosol optical depth products using 15 years of AERONET observations. *Atmospheric Environment* 2020; **225**.
11. Requia WJ, Amini H, Mukherjee R, Gold DR, Schwartz JD. Health impacts of wildfire-related air pollution in Brazil: a nationwide study of more than 2 million hospital admissions between 2008 and 2018. *Nat Commun* 2021; **12**(1): 6555.
12. O'Neill BC, Tebaldi C, van Vuuren DP, et al. The Scenario Model Intercomparison Project (ScenarioMIP) for CMIP6. *Geoscientific Model Development* 2016; **9**(9): 3461-82.
13. IPCC. IPCC, 2023: Climate Change 2023: Synthesis Report. Contribution of Working Groups I, II and III to the Sixth Assessment Report of the Intergovernmental Panel on Climate Change. Geneva, Switzerland: IPCC, 2023.
14. Gasparrini A. Modeling exposure-lag-response associations with distributed lag non-linear models. *Stat Med* 2014; **33**(5): 881-99.
15. Gasparrini A, Guo Y, Hashizume M, et al. Mortality risk attributable to high and low ambient temperature: a multicountry observational study. *Lancet* 2015; **386**(9991): 369-75.
16. Lechat B, Toson B, Scott H, et al. O011 Loss of sleep due to high ambient temperatures and its impact on healthy life years: Implications for global warming. *Sleep Advances* 2024; **5**(Supplement_1): A5-A.

Checklist of information that should be included in new reports of global health estimates

Item #	Checklist item	Reported
**Objectives and funding**		
1	Define the indicator(s), populations (including age, sex, and geographic entities), and time period(s) for which estimates were made.	Page 3, Table 1 and Table S1
2	List the funding sources for the work.	Page 25
**Data Inputs**		
For all data inputs from multiple sources that are synthesized as part of the study:		
3	Describe how the data were identified and how the data were accessed.	Page 11
4	Specify the inclusion and exclusion criteria. Identify all ad-hoc exclusions.	Page 11
5	Provide information on all included data sources and their main characteristics. For each data source used, report reference information or contact name/institution, population represented, data collection method, year(s) of data collection, sex and age range, diagnostic criteria or measurement method, and sample size, as relevant.	Page 11, 12 and data availability statement
6	Identify and describe any categories of input data that have potentially important biases (e.g., based on characteristics listed in item 5).	Localization bias is explained page 11<br>Short sleep bias, page 11
For data inputs that contribute to the analysis but were not synthesized as part of the study:		
7	Describe and give sources for any other data inputs.	Data availability statements
For all data inputs:		
8	Provide all data inputs in a file format from which data can be efficiently extracted (e.g., a spreadsheet rather than a PDF), including all relevant meta-data listed in item 5. For any data inputs that cannot be shared because of ethical or legal reasons, such as third-party ownership, provide a contact name or the name of the institution that retains the right to the data.	Data availability statements
**Data analysis**		
9	Provide a conceptual overview of the data analysis method. A diagram may be helpful.	Figure 3 and Figure 5
10	Provide a detailed description of all steps of the analysis, including mathematical formulae. This description should cover, as relevant, data cleaning, data pre-processing, data adjustments and weighting of data sources, and mathematical or statistical model(s).	Page 12, 13, 14, 15 and 16
11	Describe how candidate models were evaluated and how the final model(s) were selected.	Page 12
12	Provide the results of an evaluation of model performance, if done, as well as the results of any relevant sensitivity analysis.	Page 4
13	Describe methods for calculating uncertainty of the estimates. State which sources of uncertainty were, and were not, accounted for in the uncertainty analysis.	Page 12, 13, 14, 15 and 16
14	State how analytic or statistical source code used to generate estimates can be accessed.	Wellbeing and productivity models are available on GitHub (see code availability statement)
**Results and Discussion**		
15	Provide published estimates in a file format from which data can be	Estimate and 95%CI are

	efficiently extracted.	provided in the GitHub files
**16**	Report a quantitative measure of the uncertainty of the estimates (e.g. uncertainty intervals).	Estimate and 95%CI are provided in the GitHub files
**17**	Interpret results in light of existing evidence. If updating a previous set of estimates, describe the reasons for changes in estimates.	Page 7
**18**	Discuss limitations of the estimates. Include a discussion of any modelling assumptions or data limitations that affect interpretation of the estimates.	Page 7 and 8 discusses biases in the sample

This checklist should be used in conjunction with the GATHER statement and Explanation and Elaboration document, found on gather-statement.org
