# Supplementary material for: Global warming may increase the burden of obstructive sleep apnea
Source: Nat Commun. 2025 Jun 16;16:5100. doi: 10.1038/s41467-025-60218-1 (PMC12170837; doi:10.1038/s41467-025-60218-1)
Supplement: Supplementary file 2 — Reporting Summary [file 41467_2025_60218_MOESM2_ESM.pdf]

nature portfolio

Corresponding author(s): Dr Bastien Lechat

Last updated by author(s): Apr 10, 2025

Reporting Summary

Nature Portfolio wishes to improve the reproducibility of the work that we publish. This form provides structure for consistency and transparency in reporting. For further information on Nature Portfolio policies, see our Editorial Policies and the Editorial Policy Checklist.

Statistics

For all statistical analyses, confirm that the following items are present in the figure legend, table legend, main text, or Methods section.

n/a	Confirmed	
☐	☒	The exact sample size (n) for each experimental group/condition, given as a discrete number and unit of measurement
☐	☒	A statement on whether measurements were taken from distinct samples or whether the same sample was measured repeatedly
☐	☒	The statistical test(s) used AND whether they are one- or two-sided<br>*Only common tests should be described solely by name; describe more complex techniques in the Methods section.*
☐	☒	A description of all covariates tested
☐	☒	A description of any assumptions or corrections, such as tests of normality and adjustment for multiple comparisons
☐	☒	A full description of the statistical parameters including central tendency (e.g. means) or other basic estimates (e.g. regression coefficient) AND variation (e.g. standard deviation) or associated estimates of uncertainty (e.g. confidence intervals)
☐	☒	For null hypothesis testing, the test statistic (e.g. F, t, r) with confidence intervals, effect sizes, degrees of freedom and P value noted<br>*Give P values as exact values whenever suitable.*
☒	☐	For Bayesian analysis, information on the choice of priors and Markov chain Monte Carlo settings
☒	☐	For hierarchical and complex designs, identification of the appropriate level for tests and full reporting of outcomes
☒	☐	Estimates of effect sizes (e.g. Cohen's d, Pearson's r), indicating how they were calculated

Our web collection on statistics for biologists contains articles on many of the points above.

Software and code

Policy information about availability of computer code

Data collection	This study was a retrospective analysis of 125,295 users who registered to use an under-mattress sleep sensor (Withings Sleep Analyzer) between January 2020 and September 2023. De-identified data were provided by Withings through a collaboration agreement between Withings and Flinders University. The commercial partner had no input into the design, analysis or write up of the manuscript and did not provide any funding for this investigator-initiated study.<br><br>Weather (temperature, humidity) variables were extracted from the fifth generation of European Reanalysis (ERA5) dataset using Copernicus Climate Change Service. We extracted fine particulate matter (aerodynamic diameter <2.5 µm) concentration from the ECMWF Atmospheric Composition Reanalysis 4 model as a measure of air quality.<br><br>We used the climate scenarios CMIP6 from the 2021 sixth assessment report (AR6) of the Intergovernmental Panel on Climate Change (IPCC), also downloaded the models through the Copernicus Data Store.<br><br>Country by country informations on population, gross domestic product per capita, life expectancy were downloaded from the worldbank (https://data.worldbank.org/indicator/NY.GDP.PCAP.CD) and from the United Nations website (https://population.un.org/wpp/). Information of socio-development index for each country was extracted from the Global Burden of Disease were extracted from the 2019 estimates.<br><br>Country by country rates of motor vehicle accident and associated years of life lost were extracted from the Global burden of disease (10.1136/injuryprev-2019-043302)
Data analysis	The models for this analysis were implemented in the R programming language (version 4.3.3), using the dlnm [1] version 2.4.7 and gnm

Data analysis

packages [2] version 1.1-5. Python (version 3.11), Pandas (version 2.2.1), xarray (v2024.2.0) and MetPy (version 1.6.2) were used for data cleaning and weather extraction/calculations.

[1]. Gasparrini A. Distributed Lag Linear and Non-Linear Models in R: The Package dlnm. J Stat Softw 2011; 43(8): 1-20.
[2]. Turner H, Firth D, Ripley B, Venables B, Bates DM, Maechler M. gnm: Generalized Nonlinear Models. (Version 1.1-5) [Computer software]. . In: CRAN., editor. Retrieved from https://CRANR-projectorg/package=gnm; 2023.

Code availability: Additionnal code to reproduce the figures, wellbeing projections and economic projection are all available on GitHub: https://github.com/bastienlechat/OSA_climate.

For manuscripts utilizing custom algorithms or software that are central to the research but not yet described in published literature, software must be made available to editors and reviewers. We strongly encourage code deposition in a community repository (e.g. GitHub). See the Nature Portfolio guidelines for submitting code & software for further information.

Data

Policy information about availability of data

All manuscripts must include a data availability statement. This statement should provide the following information, where applicable:

- Accession codes, unique identifiers, or web links for publicly available datasets
- A description of any restrictions on data availability
- For clinical datasets or third party data, please ensure that the statement adheres to our policy

Deidentified raw data that support the findings of this study, including individual data, are available from the corresponding author upon request subject to ethical and data custodian (Withings) approval. ERA5 weather data and climate model projections are freely available from the Copernicus data store (https://cds.climate.copernicus.eu/). Information on country GDP, rate of motor vehicle accident, and population number are freely available from the world bank (https://www.worldbank.org/ext/en/home), the UN world population prospect (https://population.un.org/wpp/), and the global burden of disease (https://www.healthdata.org/research-analysis/gbd). Exposure-response curve between temperature and OSA risk ratio derived in this study are available on GitHub: https://github.com/bastienlechat/OSA_climate. Wellbeing and economic projection modelling for each country is also available on GitHub: https://github.com/bastienlechat/OSA_climate.

Research involving human participants, their data, or biological material

Policy information about studies with human participants or human data. See also policy information about sex, gender (identity/presentation), and sexual orientation and race, ethnicity and racism.

Reporting on sex and gender

We have use sex throughout the manuscript. No information on gender was available.

Reporting on race, ethnicity, or other socially relevant groupings

All users in this study were prompted to enter their age and sex. Participants were geo-localized to the closest largest city in each time-zone within a country (if multiple time-zones were present) or the largest city if there was only a single time-zone within a country.

Population characteristics

Data were acquired from 125,295 who registered to use an under-mattress sleep sensor (Withings Sleep Analyzer) between January 2020 and September 2023. Participants had a mean of ~500 nights of sleep recordings. Country by country demographics information is available in Table S1

Recruitment

This is a retrospective analysis of users that voluntarily used their devices (under-mattress sleep sensor or a smartwatch) between January 2020 and September 2023.

Ethics oversight

The study was approved by the Flinders University Human Research Ethics Committee (Project number: 4291).

Note that full information on the approval of the study protocol must also be provided in the manuscript.

Field-specific reporting

Please select the one below that is the best fit for your research. If you are not sure, read the appropriate sections before making your selection.

☒ Life sciences ☐ Behavioural & social sciences ☐ Ecological, evolutionary & environmental sciences

For a reference copy of the document with all sections, see nature.com/documents/nr-reporting-summary-flat.pdf

Life sciences study design

All studies must disclose on these points even when the disclosure is negative.

Sample size

This is a retrospective analysis of a consumer-database hence no sample size calculations were performed and we used all available data.

Data exclusions

Participants were required to used their device regularly and have a minimum of 28 nights of data and an average of at least 4 sleep recording nights per week to be included in the analysis. We used different inclusion/exclusions criteria in sensitivity analysis which did not change any of the main findings

Replication	The association between temperature and OSA was cross-validated across many different countries, which showed consistent effects.
Randomization	Not applicable
Blinding	Not applicable

Reporting for specific materials, systems and methods

We require information from authors about some types of materials, experimental systems and methods used in many studies. Here, indicate whether each material, system or method listed is relevant to your study. If you are not sure if a list item applies to your research, read the appropriate section before selecting a response.

Materials & experimental systems

n/a	Involved in the study	
☒	☐	Antibodies
☒	☐	Eukaryotic cell lines
☒	☐	Palaeontology and archaeology
☒	☐	Animals and other organisms
☒	☐	Clinical data
☒	☐	Dual use research of concern
☒	☐	Plants

Methods

n/a	Involved in the study	
☒	☐	ChIP-seq
☒	☐	Flow cytometry
☒	☐	MRI-based neuroimaging

Plants

Seed stocks	Not applicable
Novel plant genotypes	Not applicable
Authentication	Not applicable
